# Efficient transport kinetics of indirect excitons in van der Waals heterostructure

**DOI:** 10.1038/s41467-026-77967-2

**Published:** 2026-09-26

**Authors:** Zhiwen Zhou, W. J. Brunner, E. A. Szwed, H. Henstridge, L. H. Fowler-Gerace, L. V. Butov

**Affiliations:** Department of Physics, University of California San Diego, La Jolla, CA USA

**Keywords:** Two-dimensional materials, Two-dimensional materials

## Abstract

Exciton transport is fundamental for understanding transport phenomena in bosonic systems and for exploring excitation energy transfer in materials. Spatially indirect excitons (IXs) have long lifetimes allowing them to form quantum bosonic states and travel long distances. Van der Waals heterostructures form a new materials platform for exploring IX transport. Disordered in-plane potentials suppress IX transport due to IX localization and scattering. In this work, we found the efficient IX transport kinetics characterized by anomalously high IX mobility. The efficient IX transport kinetics is observed in the presence of substantial in-plane disorder. The anomalously high IX mobility, its disappearance with increasing temperature, and its non-monotonic dependence on density are qualitatively consistent with theoretical predictions of IX superfluidity.

## Abstract (web summary)

Here, the authors investigate the transport kinetics of indirect excitons (IXs) in a MoSe2/WSe2 van der Waals heterostructure, and observe anomalously high IX mobility, consistent with IX superfluidity.

## Introduction

Excitons, the bound pairs of electrons and holes, are composite bosons^1^. Spatially indirect excitons (IXs), also known as interlayer excitons, are formed by electrons and holes in separated layers in a heterostructure (HS)^2^. Due to the layer separation, the IX lifetimes are orders of magnitude longer than the lifetimes of spatially direct excitons (DXs)^3^. The long lifetimes allow IXs to cool below the temperature of quantum degeneracy and form quantum bosonic states^4^. Furthermore, the long IX lifetimes give an opportunity to achieve long-range exciton transport^5^. Due to these properties, IXs can be used for studying transport phenomena in quantum bosonic states and for achieving efficient excitonic energy transfer in materials.

IX transport is studied in various material platforms, including GaAs HS^5–22^, GaN HS^23^, and ZnO HS^24^. Recently, IX transport studies were started in van der Waals (vdW) HS composed of atomically thin layers of transition metal dichalcogenides (TMD). VdW TMD HS offer a unique platform for exploring exciton transport. First, excitons in vdW TMD HS, both DXs^25–27^ and IXs^28,29^, have high binding energies reaching hundreds of meV. In comparison, IX binding energies in HS formed from III–V to II–VI semiconductors are significantly lower: e.g., 3–4 meV in GaAs/AlGaAs HS^30,31^ and 30 meV in ZnO HS^32^. The high binding energies make IXs in vdW TMD HS stable at room temperature. Furthermore, the predicted superfluidity temperature that can be achieved with excitons is proportional to the exciton binding energy, and the high IX binding energies in vdW TMD HS give an opportunity to realize high-temperature superfluidity^28^. Second, IXs in periodic potentials of moiré superlattices in vdW TMD HS give an opportunity to explore the Bose–Hubbard (BH) physics^33–37^.

Exciton transport is intensively studied both with DXs in TMD monolayers^38–44^ and with IXs in vdW TMD HS^45–65^. These studies showed that disordered in-plane potentials, including disordered moiré potentials, suppress diffusive IX transport due to IX localization and scattering. In particular, even in the case of long IX lifetimes, diffusive IX transport in vdW TMD HS is characterized by low 1/*e* decay distances *d*_1/*e*_, up to a few micrometers^45–61^. Recent cw studies showed that *d*_1/*e*_ can be significantly longer^66,67^. However, cw studies do not measure the exciton transport kinetics and, therefore, do not distinguish regular diffusion from non-diffusive transport and do not determine the transport characteristics, such as the IX mobility. In particular, cw studies do not distinguish a linear growth of ${d}_{1/e}^{2}$ with time, characteristic of diffusion, from a quadratic growth of ${d}_{1/e}^{2}$ with time, characteristic of ballistic transport or drift with a nearly constant velocity. Measurements of transport kinetics *d*_1/*e*_(*t*) are needed to identify the transport regime and determine the transport characteristics.

In this work, we studied IX transport in a MoSe_2_/WSe_2_ vdW HS by time-resolved imaging that directly measures the IX photoluminescence (PL) cloud expansion and reveals the IX transport kinetics. We found efficient IX transport kinetics with a fast and quadratic growth of ${d}_{1/e}^{2}$ with time. The efficient IX transport kinetics is characterized by anomalously high IX mobility.

## Results

### Experimental results

We study MoSe_2_/WSe_2_ HS where adjacent MoSe_2_ monolayer and WSe_2_ monolayer form the separated electron and hole layers for IXs^68^. The HS details are presented in Supplementary Information (SI).

IXs are optically generated by focused laser excitation with the laser excitation energy resonant to DX in the WSe_2_ HS layer. The laser pulses have a step-like rectangular shape with 84 ns pulse width, 0.3 ns edge sharpness, and 300 ns pulse period. The IX transport is measured by time- and spatially resolved optical imaging. The measurement details are presented in SI.

**Fig. 1:**
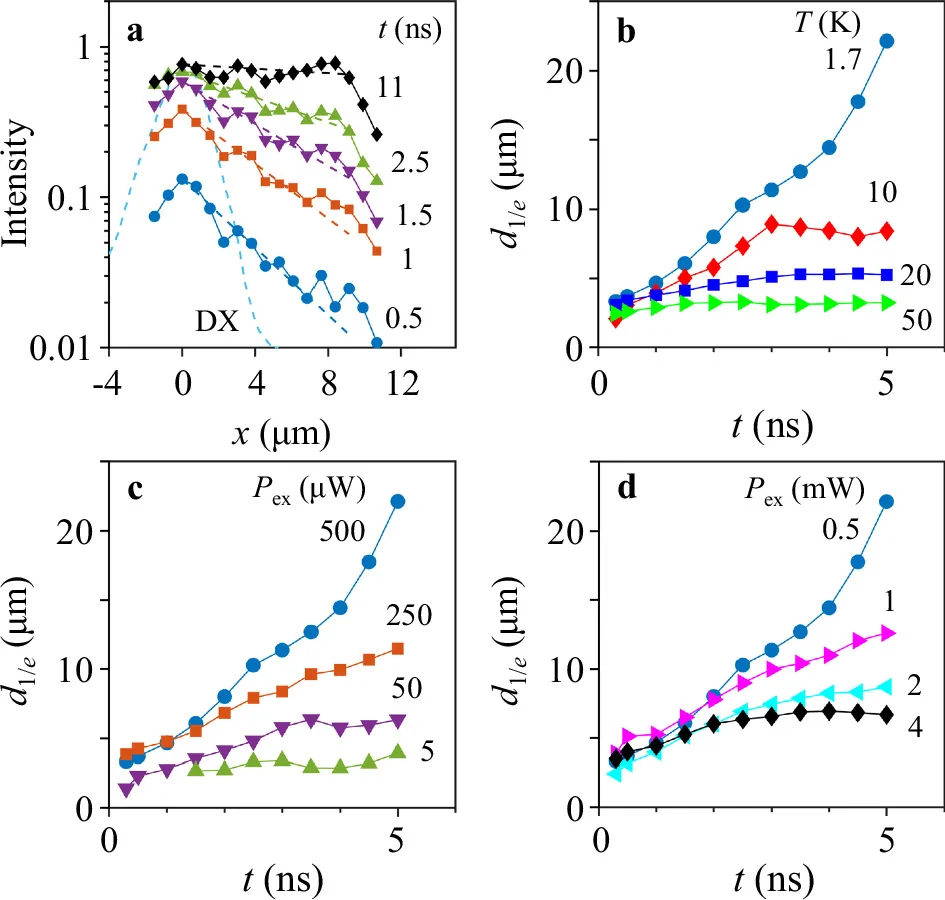
Kinetics of IX cloud expansion. **a** IX PL spatial profiles *I*(*x*) vs. time *t* after the start of the laser excitation pulse. IXs are created by the laser excitation. The excitation spot is centered at *x* = 0 and is shown by the cyan dotted line that presents the DX PL profile in MoSe_2_ monolayer and is close to the laser profile for short-range DX transport. The dashed lines show least-squares fits of the IX cloud profiles *I*(*x*, *t*) to exponential decays in the region from the excitation spot to the HS edge, *x* = 0–9 μm. The 1/*e* decay distance of the IX cloud profiles, *d*_1/*e*_ vs. *t* for different temperatures *T* (**b**) and laser excitation powers *P*_ex_ (**c**, **d**). *P*_ex_ = 0.5 mW in (**a**, **b**), *T* = 1.7 K in (**a**, **c**, **d**). The IX cloud expands, and *d*_1/*e*_ increases with time due to IX transport. The efficient IX transport kinetics is observed in a certain range of temperatures and densities, and vanishes at high temperatures (**b**) and at lower (**c**) or higher (**d**) excitation densities.

After the onset of the laser pulse, the IX cloud expands due to IX transport from the excitation spot. Figure 1a shows the kinetics of the IX signal profiles *I*(*x*, *t*). The spatial extension of the IX cloud is characterized by its 1/*e* decay distance *d*_1/*e*_(*t*) that is determined by a least-squares exponential fit to *I*(*x*, *t*) in the HS.

Figure 1b–d shows the kinetics of *d*_1/*e*_(*t*) for different temperatures *T* and laser excitation powers *P*_ex_. The enhancement of *d*_1/*e*_(*t*) with time quantifies the kinetics of the IX cloud expansion due to IX transport. The efficient IX transport kinetics with rapid *d*_1/*e*_(*t*) enhancement is observed in a certain range of temperatures and densities and vanishes at high temperatures, as shown in Fig. 1b, and at lower or higher excitation densities, as shown in Fig. 1c and Fig. 1d, respectively.

### Analysis of experimental data

For characterization of IX transport kinetics, we use two elemental approaches—in terms of diffusion (Eq. 1) and ballistic transport (Eq. 2), and an approach including drift (Eq. 3). These approaches attribute quantitative characteristics to IX transport, such as diffusivity, velocity, and mobility, that enable transport comparison at different parameters. We note that these approaches are used for the characterization of IX transport and for rough estimates of the transport characteristics; however, they do not aim to provide an accurate description of the efficient IX transport.

The advantage of the first two approaches is their simplicity. Each of them has only one fit parameter—IX diffusivity *D* or velocity *v*. However, these two approaches neglect IX drift. The third approach is more complex; however, it includes both the drift due to IX interaction and the drift due to the HS long-scale in-plane potential. Adopting the mean-field model for IX interaction^69^ allows describing IX transport, including drift, with one fit parameter—IX mobility *μ*. These approaches are outlined below.

Within the first approach, we characterize the measured IX transport by comparing it with diffusion described by the diffusion equation for IX density *n*

1 $$\frac{\partial n}{\partial t}=D\Delta n+{{\Lambda }}-\frac{n}{\tau }\,$$

where the first term describes IX diffusion with diffusivity *D*, the second IX generation by laser excitation, and the third IX recombination. The IX generation profile Λ(*x*) is given by the DX PL profile in MoSe_2_ monolayer that is close to the laser profile for the short-range DX transport.

**Fig. 2:**
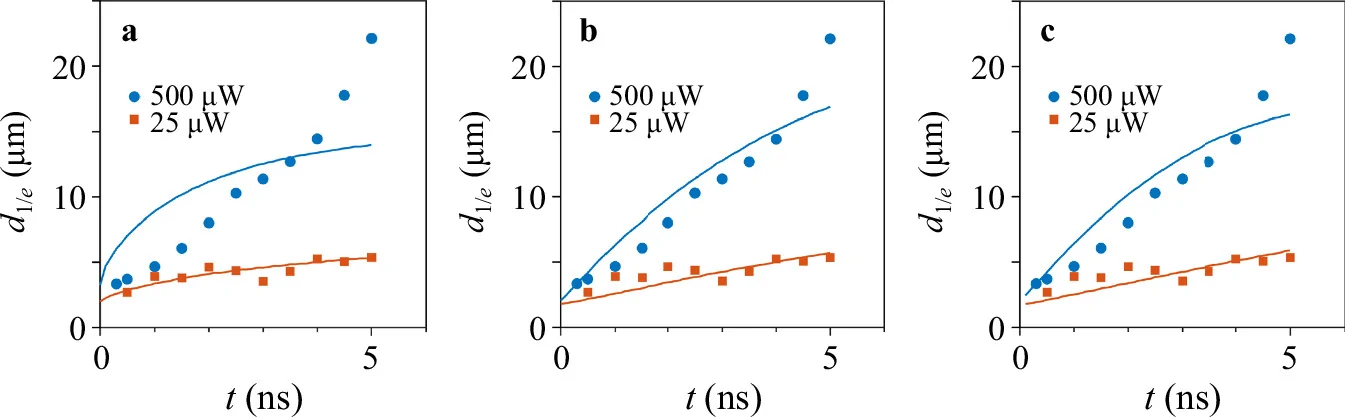
Simulations of IX cloud expansion kinetics. The measured IX cloud expansion *d*_1/*e*_(*t*) is shown by blue points for excitation power *P*_ex_ = 500 μW and by red squares for *P*_ex_ = 25 μW. The simulated *d*_1/*e*_(*t*) for diffusion (**a**), ballistic (**b**), and drift-diffusion (**c**) IX transport are shown by lines. In the simulations, the IX diffusivity *D* for diffusion (**a**), IX velocity *v* for ballistic (**b**), and IX mobility *μ* for drift-diffusion (**c**) transport are estimated by least-squares fit of the kinetics of exciton cloud expansion *d*_1/*e*_(*t*) simulated with Eqs. 1, 2, and 3, respectively, to the measured *d*_1/*e*_(*t*). The obtained *D*, *v*, and *μ* are presented in Fig. 3. The laser excitation pulse starts at *t* = 0. *T* = 1.7 K.

The IX generation by a laser excitation pulse with the rectangular step-like shape starts at *t* = 0. The IX recombination lifetime *τ* is obtained from the measurements of the decay kinetics of the spatially integrated IX signal after the excitation pulse end. The measurements of *τ* are outlined in SI. The IX diffusivity *D* is estimated by least-squares fit of the kinetics of exciton cloud expansion *d*_1/*e*_(*t*) simulated with Eq. 1 to the measured *d*_1/*e*_(*t*), see Fig. 2a.

Within the second approach, we characterize the measured IX transport by comparing it with ballistic transport described by the equation

2 $$\frac{\partial n}{\partial t}=-v\nabla n+{{\Lambda }}-\frac{n}{\tau }\,$$

where the first term describes IX ballistic transport with velocity *v*. The other terms are similar to the corresponding terms in Eq. 1. The IX velocity *v* is estimated by least-squares fit of the kinetics of exciton cloud expansion *d*_1/*e*_(*t*) simulated with Eq. 2 to the measured *d*_1/*e*_(*t*), see Fig. 2b.

Next, we consider an approach including drift. We start from the effect of IX interaction. The IX out-of-plane dipoles cause repulsive interaction between IXs, as shown by the IX studies both in GaAs HS^12,69–82^ and vdW HS^56,66,68,83,84^. As shown in Ref. ^69^, the repulsive interaction between IXs can be approximated by the mean-field formula for the IX energy shift with density δ*E* = *nu*_0_, *u*_0_ = 4*πe*^2^*d*_*z*_/*ε*, where *d*_*z*_ is the separation between the electron and hole layers, and *ε* is the dielectric constant (*d*_*z*_ ~ 0.6 nm and *ε* ~ 7.4 for the HS^85^). This mean-field model^69^ is commonly used for estimating IX densities both in GaAs HS and vdW HS, e.g., in Refs. ^6,7,11,13,15,31,46,48,56,60,62,66,69,75,82^. We use the mean-free model^69^ for density estimates in this work. We note that including correlations lowers *u*_0_ in comparison to the mean-field value, leading to a higher estimated *n* for a given δ*E*^12,77–79,81,86^. The IX repulsive interaction introduces interaction-induced drift from the origin, adding the term $\nabla \left[\mu n\nabla (n{u}_{0})\right]$ to Eq. 1, where *μ* is the IX mobility^75^.

We also include IX drift due to the in-plane potential energy landscape in the HS, *U*. The landscape originates from the variation of the local IX environment in the HS and is outlined in SI. This drift adds $\nabla \left[\mu n\nabla U\right]$ to Eq. 1^75^.

The third approach to characterize the IX transport kinetics includes both the interaction-induced drift and the drift due to the potential energy landscape in the HS and describes IX transport by the equation

3 $$\frac{\partial n}{\partial t}=\nabla \left[{D}^{{\prime} }\nabla n+\mu n\nabla (n{u}_{0}+U)\right]+{{\Lambda }}-\frac{n}{\tau }\,$$

The second term in the square brackets describes the drift due to interaction and in-plane potential energy landscape outlined above. The other terms are similar to the corresponding terms in Eq. 1 (*D* and ${D}^{{\prime} }$ are used in Eqs. 1 and 3 to avoid confusion). We use the Einstein relationship ${D}^{{\prime} }=\mu {k}_{\mathrm{B}}T$ (*k*_B_ is the Boltzmann constant) and estimate IX mobility *μ* by least-squares fit of the kinetics of exciton cloud expansion *d*_1/*e*_(*t*) simulated with Eq. 3 to the measured *d*_1/*e*_(*t*), see Fig. 2c.

**Fig. 3:**
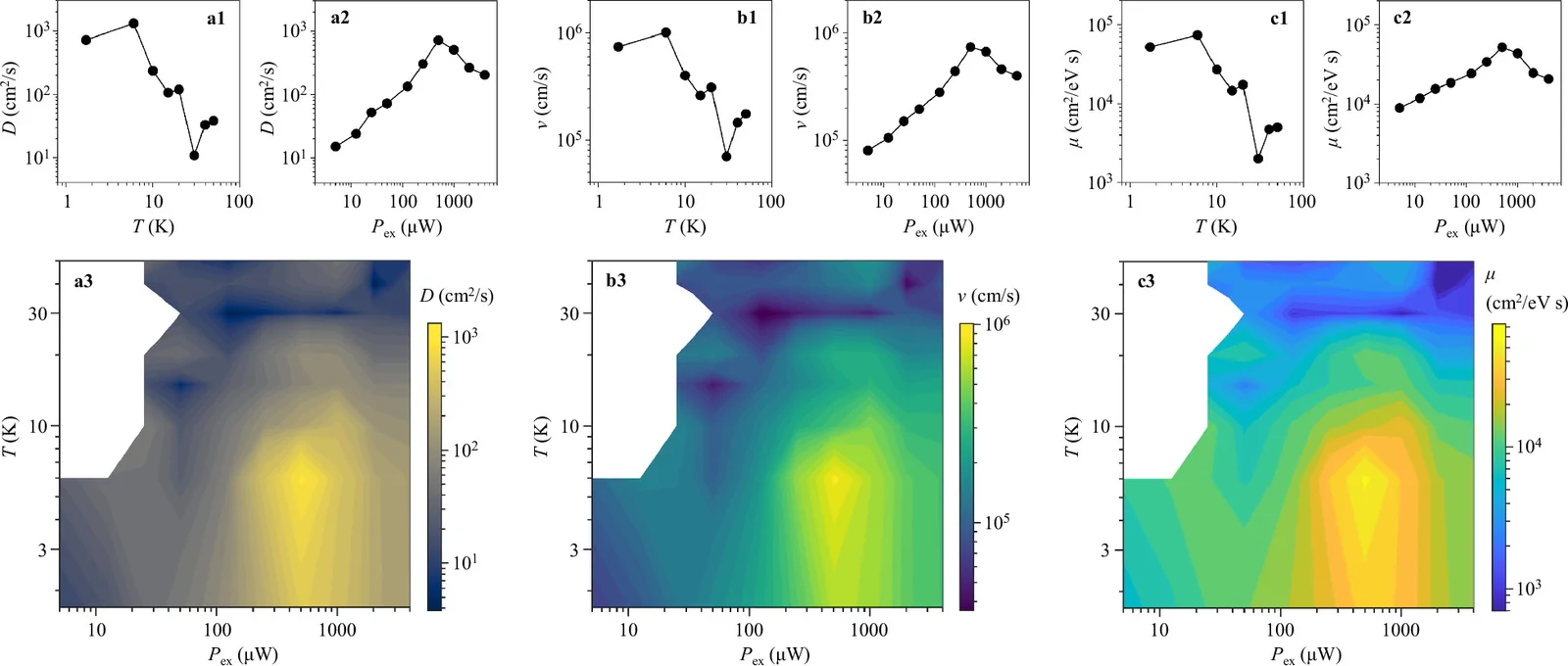
Characterization of temperature and excitation power dependence of IX transport kinetics. **a** Diffusion approach, **b** ballistic approach, and **c** drift-diffusion approach. The IX diffusivity *D* in (**a**), the IX velocity *v* in (**b**), and the IX mobility *μ* in (**c**) is estimated by least-squares fit of the kinetics of exciton cloud expansion *d*_1/*e*_(*t*) simulated with Eq. 1 in (**a**), Eq. 2 in (**b**), and Eq. 3 in (**c**), respectively, to the measured *d*_1/*e*_(*t*). **a** The estimated *D* vs. temperature *T* (a1), excitation power *P*_ex_ (a2), and *T* and *P*_ex_ (a3). **b** The estimated *v* vs. *T* (b1), *P*_ex_ (b2), and *T* and *P*_ex_ (b3). **c** The estimated *μ* vs. *T* (c1), *P*_ex_ (c2), and *T* and *P*_ex_ (c3). *P*_ex_ = 0.5 mW (a1, b1, c1), *T* = 1.7 K (a2, b2, c2).

For the HS geometry elongated along the *x* direction, as outlined in SI, the IX transport is measured along *x* and is approximated in the simulations as one-dimensional. *U*(*x*) drops ~10 meV on ~10 μm, as outlined in SI, and is approximated by the energy gradient 1 meV/μm. The obtained estimates for the IX diffusivity *D* within the diffusion approach, for the IX velocity *v* within the ballistic approach, and for the IX mobility *μ* within the drift-diffusion approach are presented in Fig. 3.

The simulations for diffusion transport show nearly linear growth of ${d}_{1/e}^{2}$ with time (nearly square-root-power growth of *d*_1/*e*_ with time); see Fig. 2a. The simulations for ballistic transport and the simulations for drift-diffusion transport show nearly quadratic growth of ${d}_{1/e}^{2}$ with time (nearly linear growth of *d*_1/*e*_ with time); see Fig. 2b, c. There are deviations from linear growth of ${d}_{1/e}^{2}(t)$ for diffusion and from quadratic growth of ${d}_{1/e}^{2}(t)$ for both ballistic and drift-diffusion transport. In particular, exciton recombination makes the growth slower.

All approaches show a similar dependence of IX transport kinetics on parameters: the efficient IX transport kinetics, characterized by high *D*, *v*, and *μ*, is observed in a certain range of temperatures and excitation densities and vanishes at high temperatures and at lower or higher excitation densities; see Fig. 3. The considered approaches quantify the temperature and excitation density dependence of the rapid *d*_1/*e*_(*t*) enhancement observed in the experiment; see Fig. 1b–d.

### Anomalously fast *d*_1/*e*_(*t*) growth

IX transport kinetics is intensively studied in vdW heterostructures^52–54,56,57,61^. The comparison of the data *d*_1/*e*_(*t*) in different studies of IX transport kinetics in vdW HS is shown in Fig. S12 in the SI. Figure S12 shows that *d*_1/*e*_(*t*) grows significantly faster for the efficient IX transport in comparison to IX transport in earlier studies of vdW HS.

### *d*_1/*e*_(*t*) growth kinetics

In earlier studies, IX diffusion or subdiffusion with ${d}_{1/e}^{2}$ growing linearly or sublinearly with time was measured^52–54,56,57,61^, see Fig. S12b. In contrast, for the efficient IX transport, ${d}_{1/e}^{2}$ grows nearly quadratically with time; see Fig. S12a, or, equivalently, *d*_1/*e*_ grows nearly linearly with time; see Fig. 2. The nearly linear growth of transport distance *d*_1/*e*_ with time corresponds to transport with a nearly constant velocity that is characteristic of ballistic transport or drift; see Fig. 2b, c.

The anomalously fast growth of *d*_1/*e*_ gives the anomalously high IX mobility *μ* shown in Fig. 3c and, in turn, the anomalously high IX mean free time *τ*_m_ ~ *μm* in the efficient IX transport regime (*m* is the IX mass in the HS, close to the free electron mass). The estimated IX mean free time for the efficient IX transport kinetics is significantly higher than in earlier studies (see SI).

The high *τ*_m_ corresponds to the high mean free path *l*_m_ ∝ *τ*_m_. The mean free path gives the range of ballistic transport, and the high *l*_m_ for the efficient IX transport kinetics indicates long-range ballistic transport.

The anomalously high IX mobility indicates suppressed IX scattering. IXs move in the HS disorder potential with the amplitude presented by the IX PL linewidth ca. 10 meV (see SI). The realization of the anomalously high IX mobility in the presence of the substantial scattering disorder is qualitatively consistent with the suppression of scattering due to superfluidity predicted for IXs in vdW HS (Ref. ^28^ and refs. therein).

### Comparison of IX transport with diffusive transport

For diffusive transport comprising both diffusion and drift-diffusion in the HS in-plane disorder potential, IX transport increases both with temperature, due to IX delocalization from local minima of the disorder potential, and with density, due to IX screening of the disorder potential and enhanced interaction-induced drift from the origin^9,12,75^. However, for the efficient IX transport kinetics, a qualitatively different behavior is observed: the efficient IX transport vanishes with temperature and vanishes with density at high densities, as shown in Fig. 3, indicating that the efficient IX transport kinetics is inconsistent with the diffusive transport.

Furthermore, both the estimated amplitude of the moiré potential^33–37^ and the disorder amplitude outlined above are high. They are significantly higher than the thermal energy at the temperatures where the efficient IX transport kinetics is observed (*k*_B_*T* ≲ 1 meV, see Fig. 3), so the IX thermal energy is insufficient for effective IX delocalization from the local minima of the in-plane potential for the diffusive IX transport^75^. They are also significantly higher than the IX interaction energy at the densities where the efficient IX transport kinetics is observed (the IX energy shift due to the interaction ~3 meV for the excitation densities corresponding to the efficient IX transport kinetics, see SI), so the IX interaction energy is insufficient for effective screening of the in-plane potential for the diffusive IX transport^12,16^. Therefore, the efficient IX transport kinetics characterized by the anomalously high IX mobility, despite the substantial in-plane disorder, is inconsistent with the diffusive transport.

### Comparison of IX transport with BH theory

The efficient IX transport kinetics characterized by the anomalously high IX mobility, despite the substantial in-plane disorder, is qualitatively consistent with the theoretically predicted superfluidity. The BH model predicts the superfluid phase for the number of bosons per lattice site *N* ~ 1/2 and the insulating phase, such as the Mott insulator and the Bose glass, for *N* ~ 0 and *N* ~ 1^87^. The IX transport enhancement followed by the suppression with density, see Fig. 3, is qualitatively consistent with this prediction. The rough estimates also indicate a qualitative agreement with the BH model: for the efficient IX transport kinetics at *P*_ex_ ~ 500 μW, the energy shift δ*E* ~ 3 meV (SI); for this energy shift, the mean-field estimate for the IX density^69^ gives *n* ~ 2 × 10^11^ cm^−2^ and *N* ~ 1/2, as outlined in SI. The density ranges for both the measured efficient IX transport and superfluidity in the BH model are broad, and these rough estimates provide qualitative agreement between the measurements and the model. The efficient IX transport kinetics vanishes above *T* ~ 10 K; see Fig. 3. This is also qualitatively consistent with the BH theory prediction for superfluidity. A comparison with the BH model is outlined in SI.

The approaches used for the characterization of IX transport in this paper outline the range of parameters where efficient IX transport kinetics is observed; they do not aim to provide an accurate description of the efficient IX transport kinetics. In particular, developing a theory of superfluid transport kinetics is beyond the scope of this paper.

In summary, we found in vdW TMD HS efficient IX transport kinetics characterized by anomalously high IX mobility. The efficient IX transport kinetics is observed in the presence of substantial in-plane disorder. The anomalously high IX mobility, its disappearance with increasing temperature, and its non-monotonic dependence on density are qualitatively consistent with theoretical predictions of IX superfluidity.

## Methods

### Heterostructure

The MoSe_2_/WSe_2_ HS was assembled using the dry-transfer peel technique^88^. The heterostructure details are presented in SI.

### Optical measurements

Excitons were generated by a semiconductor laser with the excitation energy *E*_ex_ = 1.689  eV, resonant to DX in the WSe_2_ HS layer. The laser pulses had a step-like rectangular shape with 84 ns pulse width, 0.1 ns pulse rise time, 0.3 ns pulse fall time, and 300 ns pulse period. The ~220 ns off time exceeded the IX lifetime and was sufficient for a substantial decay of the IX signal. The IX transport was measured by time- and spatially resolved optical imaging using an avalanche photodiode (APD). The APD was moved in the plane of the optical image to measure the IX PL kinetics *I*(*x*, *t*). PL spectra were measured using a spectrometer and a liquid-nitrogen-cooled charge-coupled device. The experiments were performed in a variable-temperature ^4^He cryostat. The measurement details are presented in SI. All phenomena presented in this work are reproducible after multiple cooling down to 2 K and warming up to room temperature.

## Supplementary information

Supplementary Information

Transparent Peer Review File

## Data Availability

Source data are provided within figshare https://doi.org/10.6084/m9.figshare.33000467.
