# Supplementary material for: Efficient transport kinetics of indirect excitons in van der Waals heterostructure
Source: Nat Commun. 2026 Sep 26;17:11155. doi: 10.1038/s41467-026-77967-2 (PMC13710747; doi:10.1038/s41467-026-77967-2)
Supplement: Supplementary file 1 [file 41467_2026_77967_MOESM1_ESM.pdf]

Supporting Information for
Efficient transport kinetics of indirect excitons in van der Waals heterostructure

Zhiwen Zhou,[1] W. J. Brunner,[1] E. A. Szwed,[1] H. Henstridge,[1] L. H. Fowler-Gerace,[1] and L. V. Butov[1]

[1]*Department of Physics, University of California San Diego, La Jolla, CA 92093, USA*

Heterostructure

The vdW $MoSe_2/WSe_2$ HS (Fig. S1a) was assembled using the dry-transfer peel technique [1]. The HS manufacturing details are described in Ref. [2], where the same HS was used for cw studies of IXs. The thickness of the bottom hBN layer is ∼ 40 nm, the thickness of the top hBN layer is ∼ 30 nm. The $MoSe_2$ monolayer is on top of the WSe_2 monolayer. The long WSe_2 and $MoSe_2$ edges (Fig. S1b) enable a rotational alignment between the WSe_2 and $MoSe_2$ monolayers. The twist angle between the monolayers $\delta\theta = 1.1^\circ$ corresponding to the moiré superlattice period $b = 17$ nm agrees with the angle between $MoSe_2$ and WSe_2 edges in the HS (Fig. S1b).

The accuracies of estimating $\delta\theta$ using the long WSe_2 and $MoSe_2$ edges and using SHG are comparable. We do not use SHG for additional estimates of $\delta\theta$ since the intense excitation pulses in SHG measurements may cause a deterioration of the HS and may suppress the efficient IX transport. The moiré potentials can be affected by atomic reconstruction [3–5] and by disorder and may vary over the HS area.

The IX g-factor is determined by the HS stacking [6, 7]. The IX g-factor ∼ −16 in the $MoSe_2/WSe_2$ HS corresponds to H stacking.

Figure S1b presents a microscope image showing the layer pattern of the HS. The layer boundaries are indicated. The hBN layers cover the entire areas of $MoSe_2$ and WSe_2 layers. There was a narrow multilayer graphene electrode on the top of the HS around $x = 2$ μm for $y = 0$ in Fig. S1b. This electrode was detached.

So far, the efficient IX transport kinetics in vdW TMD HS was realized in one sample in this work. Other samples in other studies of vdW TMD heterostructures show diffusive IX transport characterized by significantly smaller diffusivities as outlined in the main text. The lack of the efficient IX transport kinetics likely originates from stronger disorder, which causes IX scattering and localization. When disorder is large it breaks exciton superfluidity. With lowering the disorder, superfluidity first emerges in small disconnected puddles, then the puddles grow in size, and for the sufficiently low disorder superfluid percolating through the entire sample emerges. This leads to efficient IX transport through the entire vdW TMD HS. This work demonstrates the existence of the efficient IX transport kinetics in TMD heterostructures. It is essential to study this phenomenon in other samples with different HS parameters. However, the sample statistics of other IX transport studies in vdW TMD heterostructures outlined in the main text shows that it is challenging to manufacture samples with different HS parameters, all with sufficiently small disorder. This is the subject for future works.

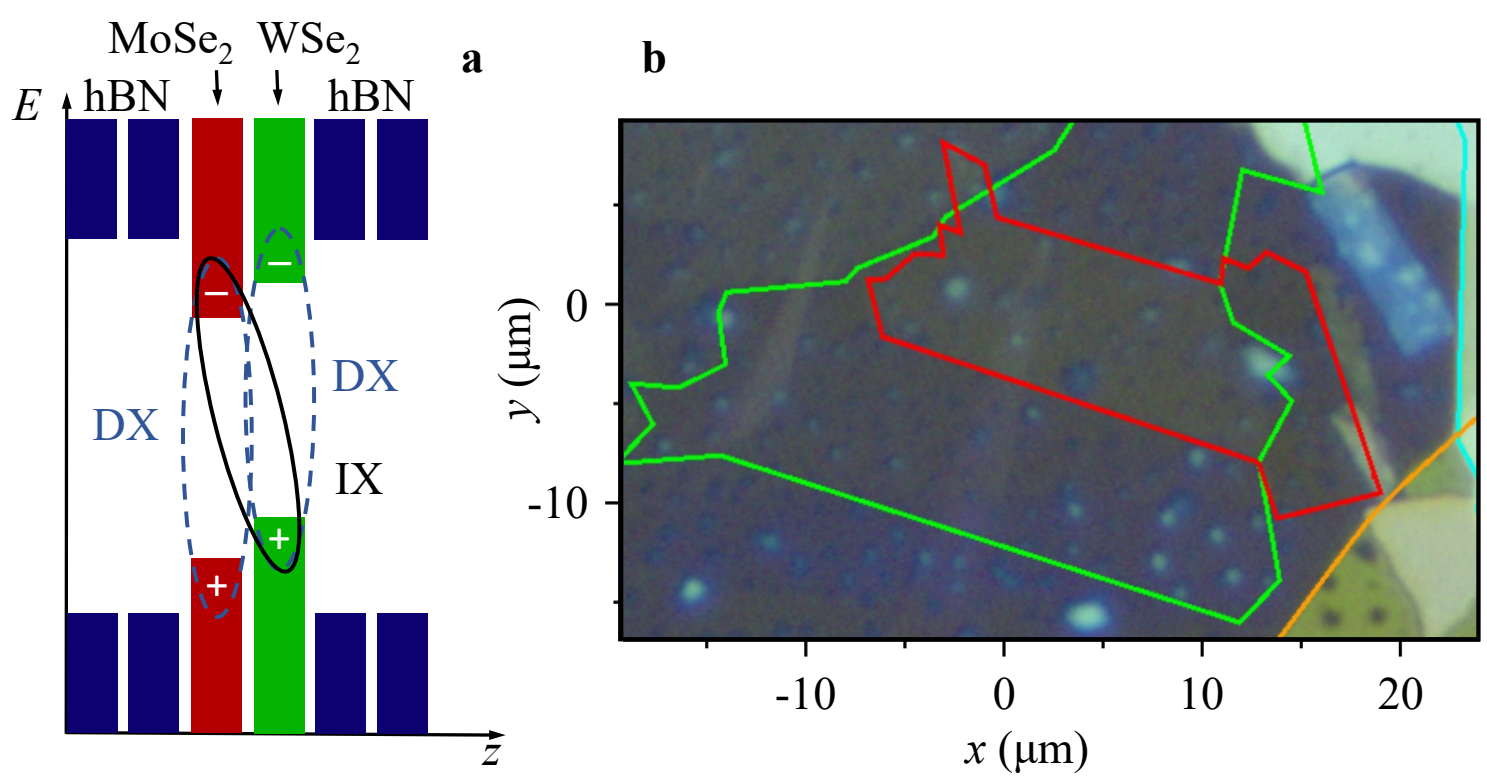

FIG. S1: $MoSe_2/WSe_2$ vdW HS. (a) Schematic energy-band diagram of the HS. The ovals indicate DXs and IX composed of an electron (–) and a hole (+). (b) A microscope image showing the HS layers. The green, red, cyan, and orange lines indicate the boundaries of WSe_2 and $MoSe_2$ monolayers and bottom and top hBN layers, respectively.

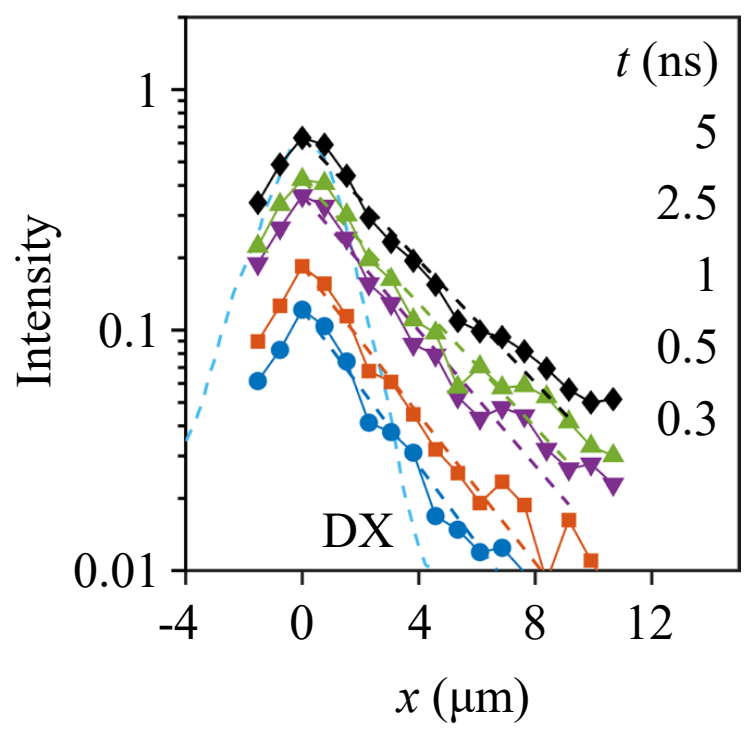

FIG. S2: IX PL spatial profiles $I(x)$ vs. time t after the start of laser excitation pulse. IXs are created by the laser excitation. The excitation spot is centered at $x = 0$ and is shown by cyan dotted line that presents the DX PL profile in $MoSe_2$ monolayer and is close to the laser profile for short-range DX transport. The dashed lines show least-squares fits of the IX cloud profiles $I(x,t)$ to exponential decays in the region from the excitation spot to the HS edge, $x = 0 - 9$ μm. $P_{ex} = 0.5$ mW, $T = 50$ K. The IX cloud expands and the $1/e$ decay distance of the IX cloud $d_{1/e}$ increases with time due to IX transport. In contrast to the data in Fig. 1a in the main text presenting $I(x,t)$ for the efficient IX transport kinetics, the enhancement of $d_{1/e}$ with time in Fig. S2 is significantly slower that is characterized, in particular, by significantly lower IX diffusivity at these parameters. The diffusivity dependence on parameters is presented in Fig. 3 in the main text.

Optical measurements

Excitons were generated by a semiconductor laser with the excitation energy $E_{ex} = 1.689$ eV resonant to DX in WSe_2 HS layer. The laser pulses had step-like rectangular shape with 84 ns pulse width, 0.1 ns pulse rise time, 0.3 ns pulse fall time (the pulses rise and fall e times within 0.1 and 0.3 ns, respectively), and 300 ns pulse period. The $\sim$ 220 ns off time exceeded the IX lifetime (Fig. S3) and was sufficient for a substantial decay of the IX signal.

The IX transport was measured by time- and spatially-resolved optical imaging using an avalanche photodiode (APD). The APD was moved in the plane of the optical image to measure the IX PL kinetics $I(x,t)$. For the image magnification x92 and the APD window diameter 100 μm, the spatial integration of the imaging setup $\sim$ 1.1 μm was substantially smaller than the IX transport distances (Fig. 1 in the main text). The APD time resolution was 0.1 ns. Both the spatial and time resolution of the time-resolved imaging setup was sufficient for the time-resolved imaging of the IX transport. The IX emission in the time-resolved imaging experiments was spectrally selected by the long-pass filter with cut-off wavelength 850 nm (1.459 eV).

PL spectra were measured using a spectrometer with a resolution of 0.2 meV and a liquid-nitrogen-cooled charge-coupled device. The experiments were performed in a variable-temperature 4He cryostat. The sample was mounted on an Attocube *xyz* piezo translation stage allowing adjusting the sample position relative to a focusing lens inside the cryostat. All phenomena presented in this work are reproducible after multiple cooling down to 2 K and warming up to room temperature.

Spatial profiles $I(x,t)$

A representative example of IX cloud expansion with time $I(x,t)$ in the regime of efficient IX transport is presented in Fig. 1a in the main text. Figure S2 shows a representative example of $I(x,t)$ in the regime of regular diffusive IX transport. In contrast to the efficient IX transport with the fast enhancement of the spatial extension of IX cloud $d_{1/e}(t)$ with time (Fig. 1a in the main text), for diffusive IX transport the enhancement of $d_{1/e}(t)$ with time is significantly slower (Fig. S2). The difference between the efficient IX transport regime and the diffusive transport regime is characterized, in particular, by the anomalously high IX diffusivity for the former and is outlined in the main text.

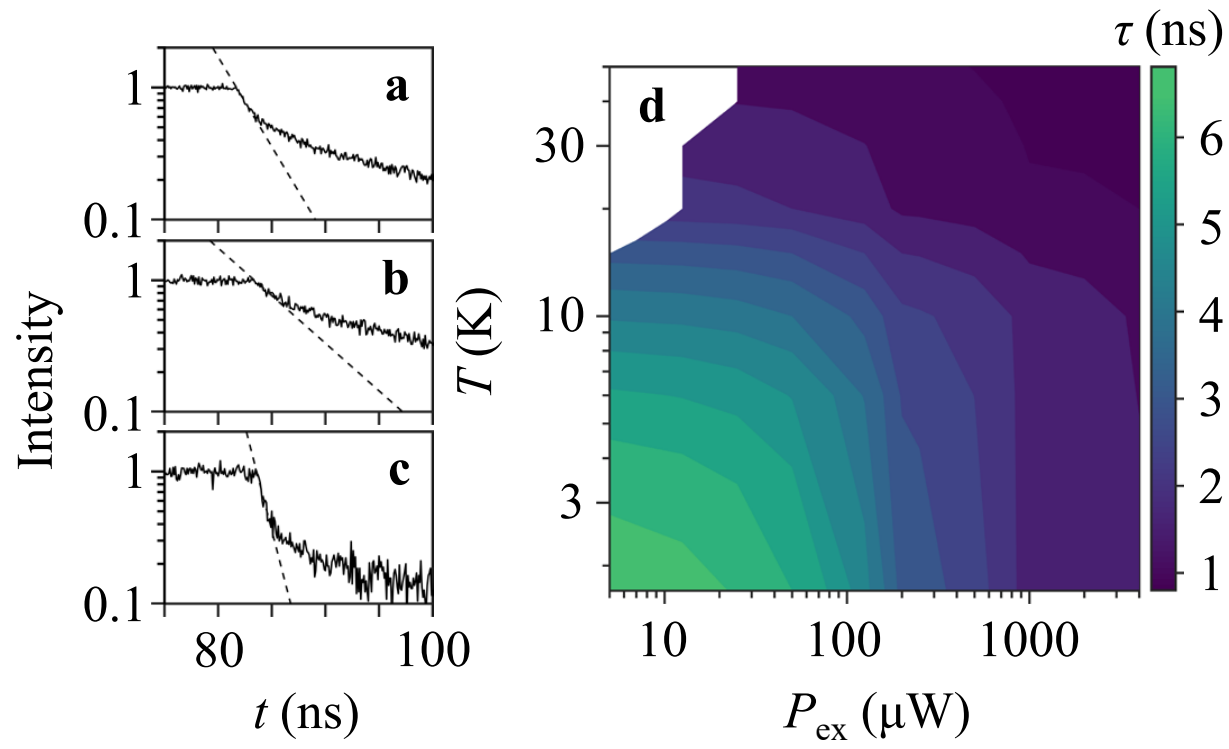

FIG. S3: Lifetime. (a-c) The decay kinetics of the spatially integrated IX signal after the excitation pulse end, $I_{\text{int}}(t)$, for different excitation powers and temperatures: $P_{\text{ex}} = 200$ μW, $T = 1.7$ K in (a), $P_{\text{ex}} = 50$ μW, $T = 1.7$ K in (b), $P_{\text{ex}} = 200$ μW, $T = 20$ K in (c). The dashed lines showing the initial slope of $\ln(I_{\text{int}}(t))$ after the excitation pulse end at $t \sim 84$ ns are used to estimate τ. (d) The IX recombination lifetime τ vs. excitation power P_{ex} and temperature T. τ is the initial decay time after the excitation pulse end.

Lifetime

The IX recombination lifetime τ was obtained from the measurements of the decay kinetics of the spatially integrated IX signal after the excitation pulse end, $I_{\text{int}}(t)$. The spatial integration minimizes the effect of IX transport on the measured kinetics and realizes the regime where the decay kinetics is governed by the IX recombination. Both increasing excitation power and temperature led to a reduction of τ (Fig. S3). These variations of τ were included in the simulations using Eqs. 1, 2, and 3 outlined in the main text, where the measured values of $\tau(P_{\text{ex}}, T)$ corresponding to the considered T and P_{ex} were used. The recombination comprises all recombination processes including Auger recombination.

The recombination rates, given by the slope of $\ln(I_{\text{int}}(t))$, reduce with time (Fig. S3a-c). The initial recombination rate after the excitation pulse end, τ^{-1}, corresponds to the IX density during the pulse. Since IX transport during the pulse is considered in the experiments (Fig. 2 in the main text), the measured initial lifetime τ (Fig. S3d) is used in the simulations. For the efficient IX transport regime, the effect of lifetime variation on kinetics is rather weak and we checked that this variation leads to minor $\sim 10\%$ changes of the parameters obtained from the kinetics fits.

Simulated spatial profiles $I(x, t)$

Examples of simulated spatial profiles $I(x, t)$ for IX diffusion and ballistic transport using, respectively, Eq. 1 and Eq. 2 in the main text are shown in Fig. S4. The simulations used the spatial profile of the laser excitation spot $\Lambda(x)$, given by the measured DX PL profile in $MoSe_2$ monolayer that is close to the laser profile for short-range DX transport, and the IX lifetime τ presented in Fig. S3.

The mean-squared displacement (MSD) and $d_{1/e}$, which quantify the spatial extension of IX spatial profiles $I(x, t)$, grow with time. MSD is close to $d_{1/e}^2$ as shown by Fig. S5. The simulations for diffusion (Fig. 2a) show nearly linear growth of MSD and $d_{1/e}^2$ with time, or, equivalently, nearly square-root-power growth of $d_{1/e}$ with time. The simulations for ballistic transport (Fig. 2b) and for drift-diffusion transport (Fig. 2c) show nearly quadratic growth of MSD and $d_{1/e}^2$ with time, or, equivalently, nearly linear growth of $d_{1/e}$ with time.

There are deviations from linear growth of MSD and $d_{1/e}^2$ with time for diffusion and there are deviations from quadratic growth of MSD and $d_{1/e}^2$ with time for ballistic transport and drift-diffusion transport. In particular, exciton recombination makes the growth slower.

While $d_{1/e}^2$ and MSD are close (Fig. S5), using $d_{1/e}^2$ (or $d_{1/e}$) is more accurate for describing experimental data. In particular, in experiments, any sample has a finite size and $d_{1/e}$ accurately quantifies the expansion of spatial profiles $I(x, t)$ even when $d_{1/e}$ exceeds the sample size, however, MSD is affected by the finite sample size and cannot be used in this case.

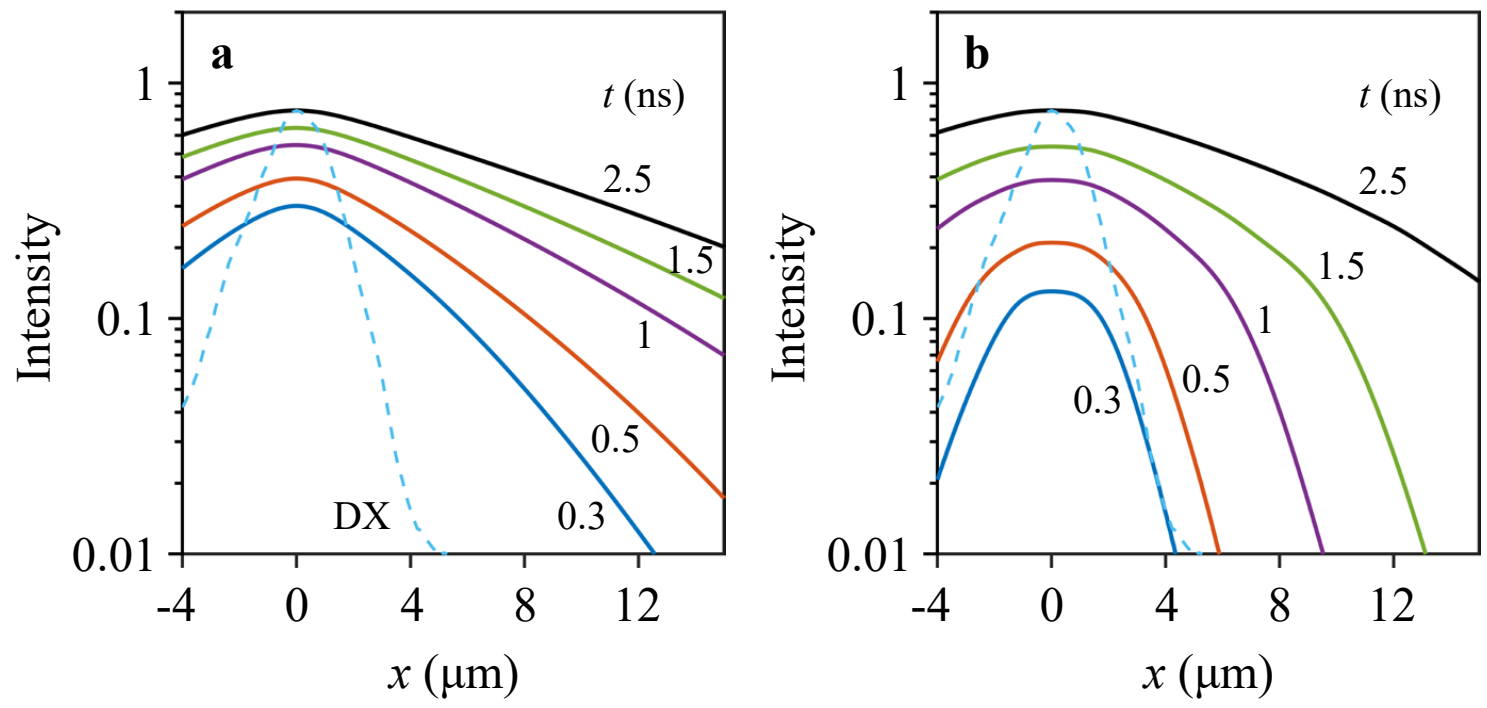

FIG. S4: Simulated $I(x,t)$. Simulated spatial profiles $I(x,t)$ for IX diffusion (a) and ballistic transport (b) using, respectively, Eq. 1 and Eq. 2 in the main text. For these simulations, the IX diffusivity $D = 720$ cm^2/s in (a), the IX velocity $v = 7.4 \cdot 10^5$ cm/s in (b), and the IX lifetime $\tau = 2.7$ ns in (a,b). The dotted lines show the spatial profile of the laser excitation spot $\Lambda(x)$. The excitation pulse starts at $t = 0$.

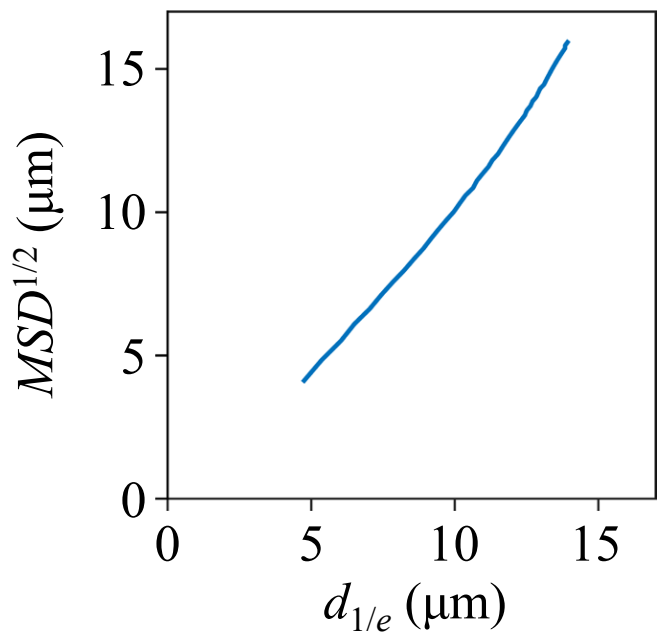

FIG. S5: Comparison of MSD and $d_{1/e}$ for $I(x,t)$ in Fig. S4a. $d^2_{1/e}$ and MSD are close.

On analysis of $d_{1/e}(t)$

In the main text, the IX diffusivity D is estimated by least-squares fit of the kinetics of exciton cloud expansion $d_{1/e}(t)$ simulated with Eq. 1 to the measured $d_{1/e}(t)$ in the time range $t = 0 - 5$ ns (Fig. 2a), and the IX velocity v is estimated by least-squares fit of the kinetics of exciton cloud expansion $d_{1/e}(t)$ simulated with Eq. 2 to the measured $d_{1/e}(t)$ in the time range $t = 0 - 5$ ns (Fig. 2b). The results are similar for the fits made in different time ranges. Figure S6 shows the fits similar to the fits in Fig. 2 in the main text, however, for the fit range $t = 0 - 3$ ns. These fits give the values of D and v shown in Fig. S7. The fits over $t = 0 - 3$ ns give similar D and v values and their similar temperature and excitation density dependence $D(P_{\text{ex}}, T)$ and $v(P_{\text{ex}}, T)$ as the fits over $t = 0 - 5$ ns in the main text (compare Fig. S7 and Fig. 3 in the main text). Furthermore, for the efficient IX transport, the fits over $t = 0 - 3$ ns show a high deviation of the measured $d_{1/e}(t)$ from diffusion (Fig. S6a) and significantly smaller deviation of the measured $d_{1/e}(t)$ from ballistic transport (Fig. S6b), similarly to the fits over $t = 0 - 5$ ns outlined in the main text. The specifics of the fits over $t = 0 - 3$ ns is that $d_{1/e}(t)$ in this time range is within the HS area (Fig. S6).

The estimates are obtained using Eqs. 1 and 2 with constant values for D and v. During the excitation pulse, the IX density varies with time. Simulations of IX kinetics with density-dependent parameters can improve the accuracy of estimates and is the subject of future works.

On long-distance IX transport and IX mediated long-distance spin transport

The density and temperature range of the efficient IX transport kinetics characterized by the anomalously high diffusivity (Fig. 3 in the main text) and nearly quadratic growth of $d^2_{1/e}$ with time, or, equivalently, nearly linear growth of $d_{1/e}$ with time (Fig. 2 in the main text), is consistent with the density and temperature range for the long-distance

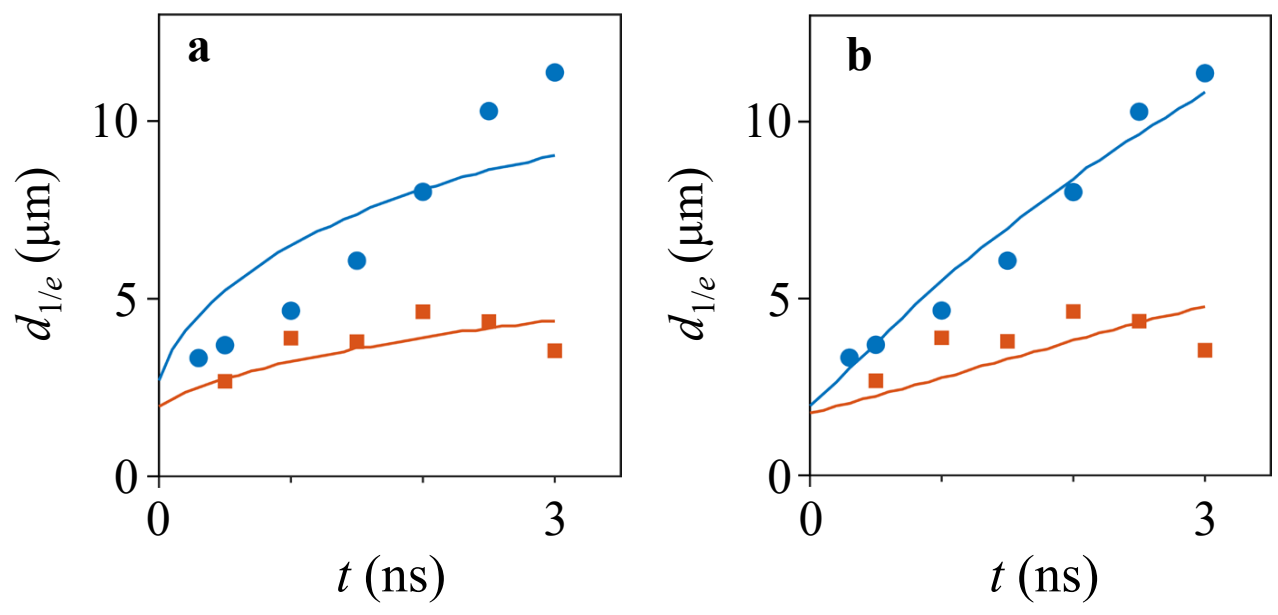

FIG. S6: Simulations of IX cloud expansion kinetics. The measured IX cloud expansion $d_{1/e}(t)$ is shown by blue points for the excitation power P_{ex} = 500 μW and by red squares for P_{ex} = 25 μW. The simulated $d_{1/e}(t)$ for IX diffusion (a) and ballistic transport (b) are shown by lines. In the simulations, the IX diffusivity D for diffusion (a) and IX velocity v for ballistic transport (b) are estimated by least-squares fit of the kinetics of exciton cloud expansion $d_{1/e}(t)$ simulated with Eq. 1 and Eq. 2 in the main text, respectively, to the measured $d_{1/e}(t)$. The simulations in Fig. S6 are similar to the simulations in Fig. 2 in the main text, however, the fit range is $t = 0 - 3$ ns in Fig. S6 and $t = 0 - 5$ ns in Fig. 2 in the main text. The estimates for D and v obtained with the simulations with the fit range $t = 0 - 3$ ns (Fig. S6) are presented in Fig. S7. The laser excitation pulse starts at $t = 0$. T = 1.7 K.

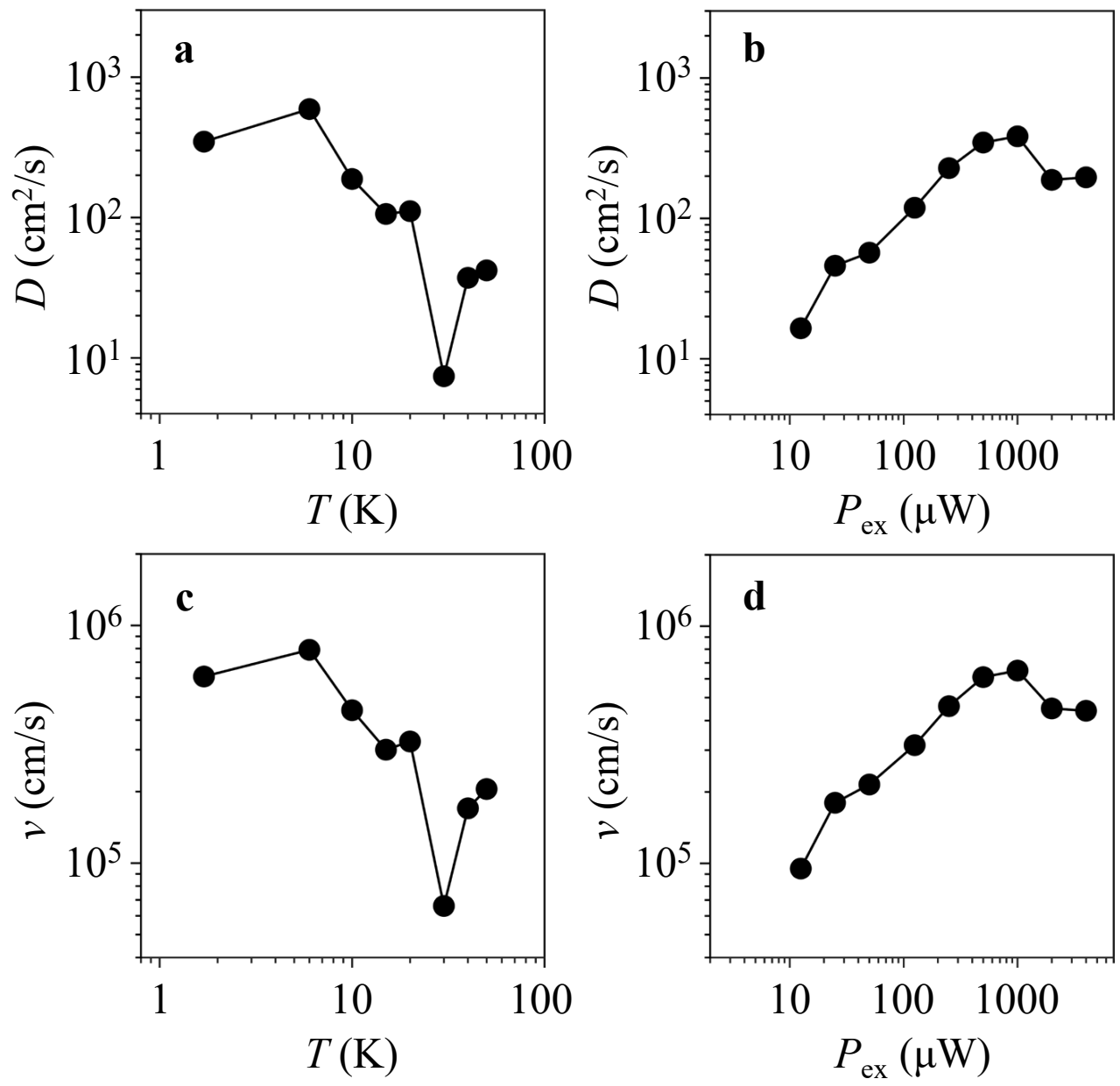

FIG. S7: Temperature and excitation power dependence of IX transport kinetics: diffusion approach (a,b) and ballistic transport approach (c,d). The IX diffusivity for diffusion and IX velocity for ballistic transport are estimated by least-squares fit of the kinetics of exciton cloud expansion $d_{1/e}(t)$ simulated with Eq. 1 and Eq 2 in the main text, respectively, to the measured $d_{1/e}(t)$. The estimates in Fig. S7 are similar to the estimates in Fig. 3 in the main text, however, the fit range is $t = 0 - 3$ ns in Fig. S7 and $t = 0 - 5$ ns in Fig. 3 in the main text. (a,b) The estimated IX diffusivity D vs. temperature (a) and excitation power (b). (c,d) The estimated IX velocity v vs. temperature (c) and excitation power (d). P_{ex} = 0.5 mW (a,c), T = 1.7 K (b,d).

IX transport and the IX mediated long-distance spin transport in cw studies of IXs in vdW TMD HS [2, 8]. This indicates that the latter phenomena originate from the efficient IX transport kinetics.

IX interaction

The IX out-of-plane dipoles cause repulsive interaction between IXs and, as shown in Ref. [9], the repulsive interaction between IXs can be approximated by the mean-field formula for the IX energy shift with density $\delta E = nu_0$, $u_0 = 4\pi e^2 d_z/\varepsilon$, where d_z is the separation between the electron and hole layers and ε is the dielectric constant

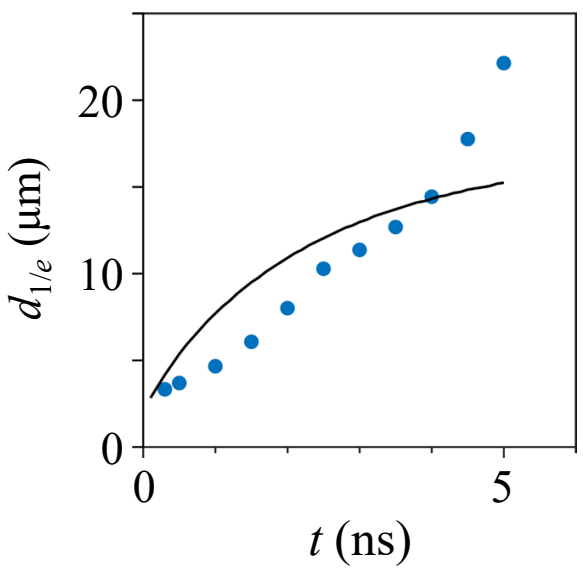

FIG. S8: Simulations of IX cloud expansion kinetics due to diffusion and interaction-induced drift. The measured IX cloud expansion $d_{1/e}(t)$ is shown by blue points for $P_{\rm ex} = 500$ μW and $T = 1.7$ K. The simulated $d_{1/e}(t)$ for IX transport due to diffusion and interaction-induced drift is shown by line. The simulations use Eq. 1 with the additional term describing the interaction-induced drift as outlined in the main text and SI. The laser excitation pulse starts at $t = 0$.

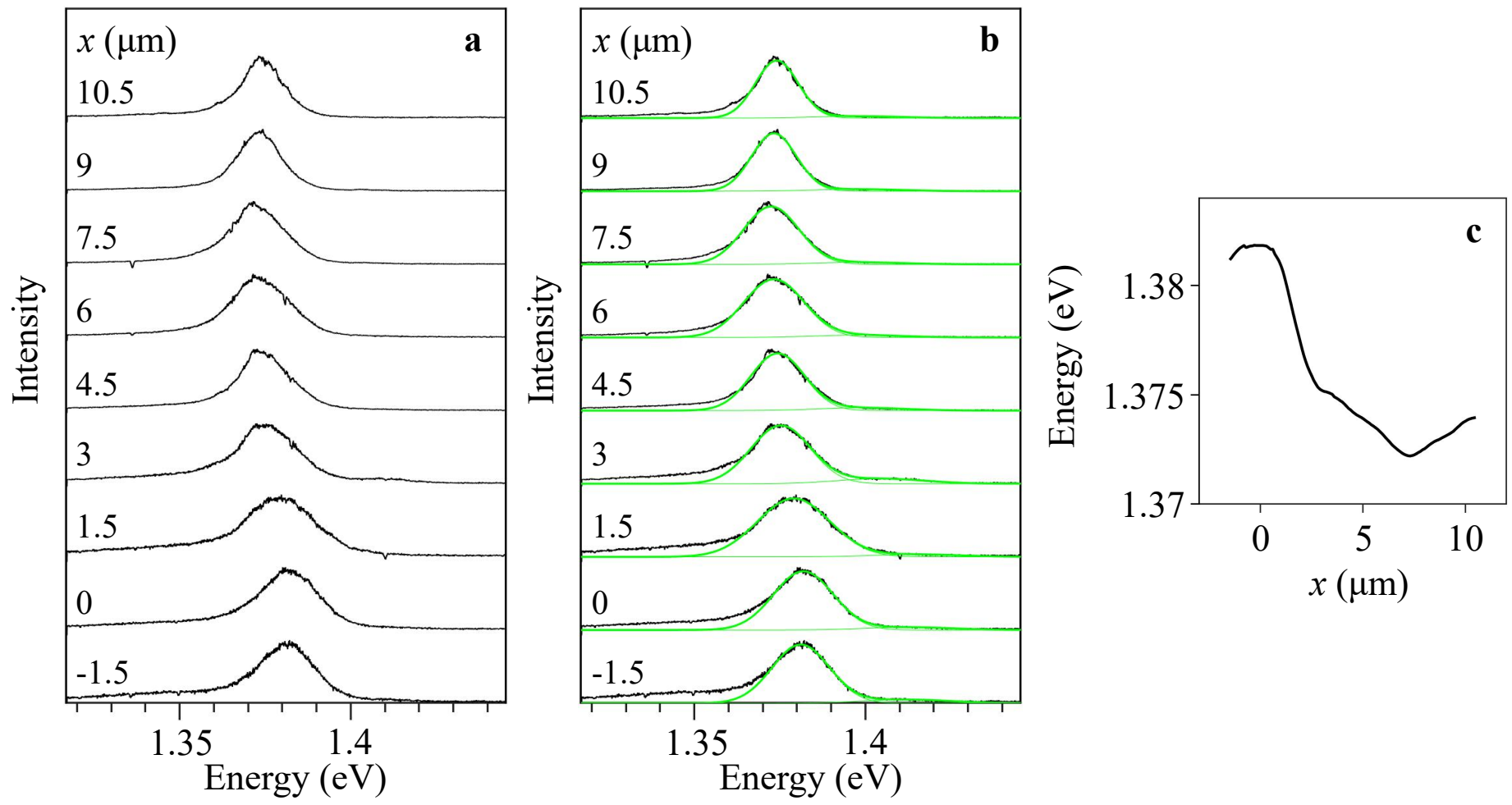

FIG. S9: Potential energy landscape. (a) IX spectra vs. position x along the IX path. (b) Same spectra with the gaussian fits shown by the green lines. (c) Position dependence of the IX energy $U(x)$. The laser excitation is defocused to ~ 25 μm spot covering the entire HS, the power of this defocused excitation is ~ 5 mW. $T = 4.2$ K.

($d_z \sim 0.6$ nm and $\varepsilon \sim 7.4$ for the HS [10]). Including correlations beyond this mean-field model lowers u_0 in comparison to the mean-field value, leading to a higher estimated n for a given δE as outlined in the main text. The IX repulsive interaction introduces interaction-induced drift from the origin adding the term $\nabla[\mu n \nabla(n u_0)]$ to Eq. 1 [11]. For the IX mobility μ estimated by the Einstein relation $\mu = D_0/k_B T$, IX diffusion with interaction-induced drift can be approximately described as diffusion with the enhanced diffusivity $D = D_0(1 + n u_0/k_{\rm B}T)$, where D_0 is the diffusivity for non-interacting particles, as outlined in Ref. [12]. Similar to diffusion (Fig. 2a in the main text), $d^2_{1/e}(t)$ grows nearly linearly with time ($d_{1/e}(t)$ grows nearly $\propto t^{1/2}$) for IX diffusion with interaction-induced drift (Fig. S8).

For the efficient IX transport kinetics at $P_{\rm ex} \sim 500$ μW (Fig. 2 in the main text), the energy shift $\delta E \sim 3$ meV (Fig. S11c). For this energy shift, the mean-field estimate for the IX density [9] outlined in the main text gives $n \sim 2 \cdot 10^{11}$ cm^{-2}.

Alternatively, IX density can be roughly estimated from the light absorption $n \sim \alpha P_{\rm ex}\tau / E_{\rm ex} A_{\rm ex}$, where $A_{\rm ex}$ is the excitation area and α is the fraction of photons provided by the excitation laser that is transformed to IXs due to the light absorption and carrier relaxation. For $P_{\rm ex} \sim 500$ μW for the laser excitation pulses, $E_{\rm ex} = 1.689$ eV, $A_{\rm ex} \sim 150$ μm^2 for the IX expansion over the entire HS at this $P_{\rm ex}$ (Fig. 1a), $\alpha \sim 0.1$, and $\tau \sim 2.7$ ns for this $P_{\rm ex}$ (Fig. S3), the estimate from the light absorption gives $n \sim 3 \cdot 10^{11}$ cm^{-2}. This value is close to the mean-field estimate from the IX energy shift outlined above. However, the exciton density estimates based on the light absorption may be inaccurate. Therefore, we use the mean-field estimate for the IX density based on the IX energy shift [9] as outlined in the main text.

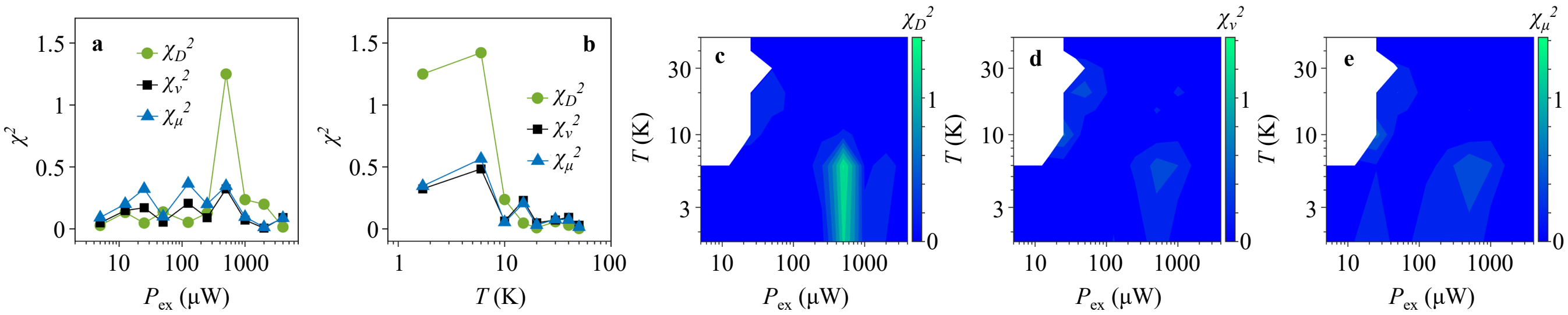

FIG. S10: Comparison of IX transport with diffusion, ballistic, and drift-diffusion transport. (a-e) The deviation of the measured IX transport kinetics from the simulated kinetics $\chi^2 = \Sigma(m_t - s_t)^2/(\nu s_t)$ vs. excitation power (a), temperature (b), and excitation power and temperature (c-e). m_t and s_t are the measured and simulated IX cloud extension $d_{1/e}$ at time t and ν is the number of the measured t values in χ^2. (a,b) Green points, black squares, and blue triangles show χ^2 deviation of the measured IX transport from the simulated diffusion, ballistic, and drift-diffusion transport, respectively. (c-e) χ^2 deviation of the measured IX transport from the simulated diffusion (c), ballistic (d), and drift-diffusion (e) transport. In the regime of the efficient IX transport (yellow region in Figs. 3a3, 3b3, and 3c3), χ^2 are large for diffusion and significantly smaller for ballistic and drift-diffusion transport. This shows that the efficient IX transport kinetics significantly deviates from the kinetics of diffusion and is close to the kinetics of both ballistic transport and drift-diffusion transport. T = 1.7 K (a), P_{ex} = 0.5 mW (b).

IX drift due to potential energy landscape

As outlined in the main text, Eq. 3 includes the term $\nabla[\mu n \nabla U]$ describing IX drift due to the in-plane potential energy landscape in the HS, U. This landscape originates from the variation of the local IX environment in the HS. The specific origin of this landscape is not essential for the IX transport modeling by Eq. 3. The measured $U(x)$ in the HS is presented in Fig. S9c. This figure shows that $U(x)$ drops $\sim$ 10 meV on $\sim$ 10 μm of the IX path from the excitation spot to the HS edge and, for the simulations using Eq. 3, $U(x)$ is approximated by the energy gradient 1 meV/μm.

The simulations in Fig. S8 include IX drift due to the IX interaction and do not include IX drift due to the in-plane potential energy landscape. These simulations show nearly square-root growth of $d_{1/e}$ with time and do not agree with the data showing nearly linear growth of $d_{1/e}$ with time. In contrast, the simulations including both drift due to the IX interaction and drift due to the in-plane potential energy landscape in the HS show nearly linear growth of $d_{1/e}$ with time (Fig. 2c) and agree with the data.

Deviation of IX transport from diffusion, ballistic, and drift-diffusion transport

We compared the deviation of IX transport kinetics from the simulated kinetics of diffusion (Fig. 2a), ballistic (Fig. 2b), and drift-diffusion (Fig. 2c) transport. The deviation is quantified by $\chi^2 = \Sigma(m_t - s_t)^2/(\nu s_t)$, where m_t and s_t are the measured and simulated IX cloud extension $d_{1/e}$ at time t and ν is the number of the measured t values in χ^2 (Fig. S10). In the regime of the efficient IX transport (yellow region in Figs. 3a3, 3b3, and 3c3), χ^2 are large for diffusion (Figs. S10a,b,c) and substantially smaller for the ballistic and drift-diffusion transport (Figs. S10a,b,d,e). This confirms that the efficient IX transport kinetics significantly deviates from the simulated kinetics of diffusion and is close to the simulated kinetics of both drift-diffusion and ballistic transport.

This χ^2 test justifies the same conclusion, which can be made by the eye comparison of the measured and simulated $d_{1/e}(t)$ in Fig. 2: The data show a strong deviation of the measured efficient IX transport kinetics from the simulated kinetics of diffusion transport (the blue dots strongly deviate from the blue line in Fig. 2a) and significantly smaller deviation of the measured efficient IX transport kinetics from the simulated kinetics of both drift-diffusion and ballistic transport (the deviation of the blue dots from the blue line in Figs. 2b,c is significantly smaller).

Comparison with BH theory

As outlined in the main text, the BH model predicts the superfluid phase and the efficient IX transport kinetics, characterized by the anomalously high IX mobility despite the in-plane disorder, is qualitatively consistent with the predicted superfluidity. Below, we discuss a rough comparison of the parameters, at which the efficient IX transport kinetics is observed, with the BH model.

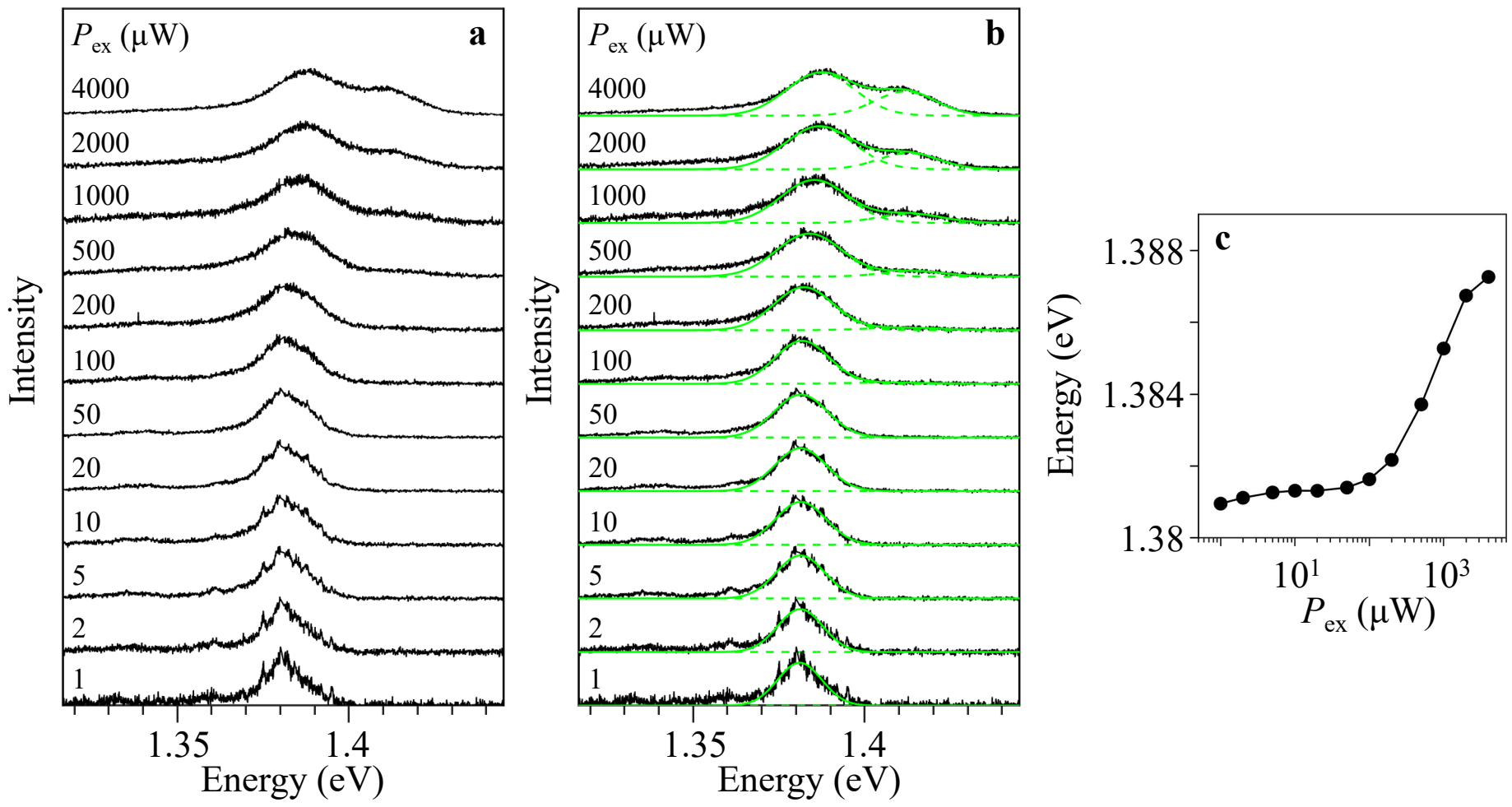

FIG. S11: IX PL spectra. (a) The excitation power P_{ex} dependence of IX spectra. $T = 6$ K. (b) Same spectra with the gaussian fits shown by the dashed green lines. The sum of the gaussians shown by the dashed green lines is shown by the solid green line. (c) P_{ex} dependence of the IX energy.

The BH model predicts the superfluid phase for the number of bosons per lattice site $N \sim 1/2$ and the insulating phase, such as the Mott insulator and the Bose glass, for $N \sim 0$ and $N \sim 1$ [13]. The IX transport enhancement followed by the suppression with density (Fig. 3 in the main text) is qualitatively consistent with this prediction. The rough estimates also indicate a qualitative agreement with the BH model: For the efficient IX transport kinetics at $P_{ex} \sim 500$ μW (Fig. 2 in the main text), the energy shift $\delta E \sim 3$ meV (Fig. S11c); For this energy shift, the mean-field estimate for the IX density, outlined in SI Section *IX interaction*, gives $n \sim 2 \cdot 10^{11}$ cm^{-2}; $N \sim 1/2$ for this density and the moiré superlattice period $b = 17$ nm, where $b \sim a/\delta\theta$ corresponds to the twist angle $\delta\theta = 1.1°$ that agrees with the angle between $MoSe_2$ and WSe_2 layers in the HS (a is the lattice constant).

The efficient IX transport is observed in a broad range of excitation powers P_{ex} (Fig. 3) and, in turn, δE given by P_{ex} (Fig. S11c) and densities n estimated from δE as outlined in SI Section *IX interaction*. This indicates that the efficient IX transport is observed in a broad range of N. The BH model also predicts superfluidity in a broad range of N [13]. The density estimates in this work are performed within the mean-field model [9] and these rough estimates give N for the efficient IX transport qualitatively consistent with N predicted for superfluidity in the Bose-Hubbard model. The broad density ranges of both the observed efficient IX transport and superfluidity in the BH model indicate that the density estimate does not have to be precise for this qualitative consistency.

The efficient IX transport vanishes above $T \sim 10$ K (Fig. 3). As outlined in the main text this is inconsistent with diffusion and, in contrast, is qualitatively consistent with superfluidity. The rough estimates, presented below, also indicate a qualitative agreement with the BH model: The BH theory predicts the critical temperature for superfluidity $T_c \sim 4\pi NJ$ [14]; For $T_c \sim 10$ K this formula gives the intersite hopping $J \sim 1.6$ K and, in turn, the moiré superlattice amplitude ~ 8 meV for $b \sim 17$ nm [15]; This value is comparable to the moiré potential amplitude for the $MoSe_2/WSe_2$ HS with H stacking [16–19] and the g-factor in the HS corresponds to H stacking [6, 7].

IX PL spectra

Figure S11 shows IX spectra at different excitation powers P_{ex}. The IX PL linewidth at low densities gives an estimate for the disorder amplitude in the HS. Both the smallest IX linewidth at the lowest densities ~ 14 meV and the IX linewidth at the excitation densities corresponding to the efficient IX transport ~ 20 meV (Fig. S11a,b) are significantly larger than the IX interaction energy at the densities where the efficient IX transport is observed ~ 3 meV (Fig. S11c). Therefore, the IX interaction energy is insufficient for effective screening of the in-plane potential for diffusive IX transport [20]. The efficient IX transport kinetics occurs despite the strong in-plane potential that is inconsistent with classical diffusive transport as outlined in the main text.

Narrow lines appearing in the IX spectrum at lower densities (Fig. S11) were attributed to localized states [8]. A higher-energy IX line appearing at high IX densities (Fig. S11) was attributed to the appearance of moiré cells with

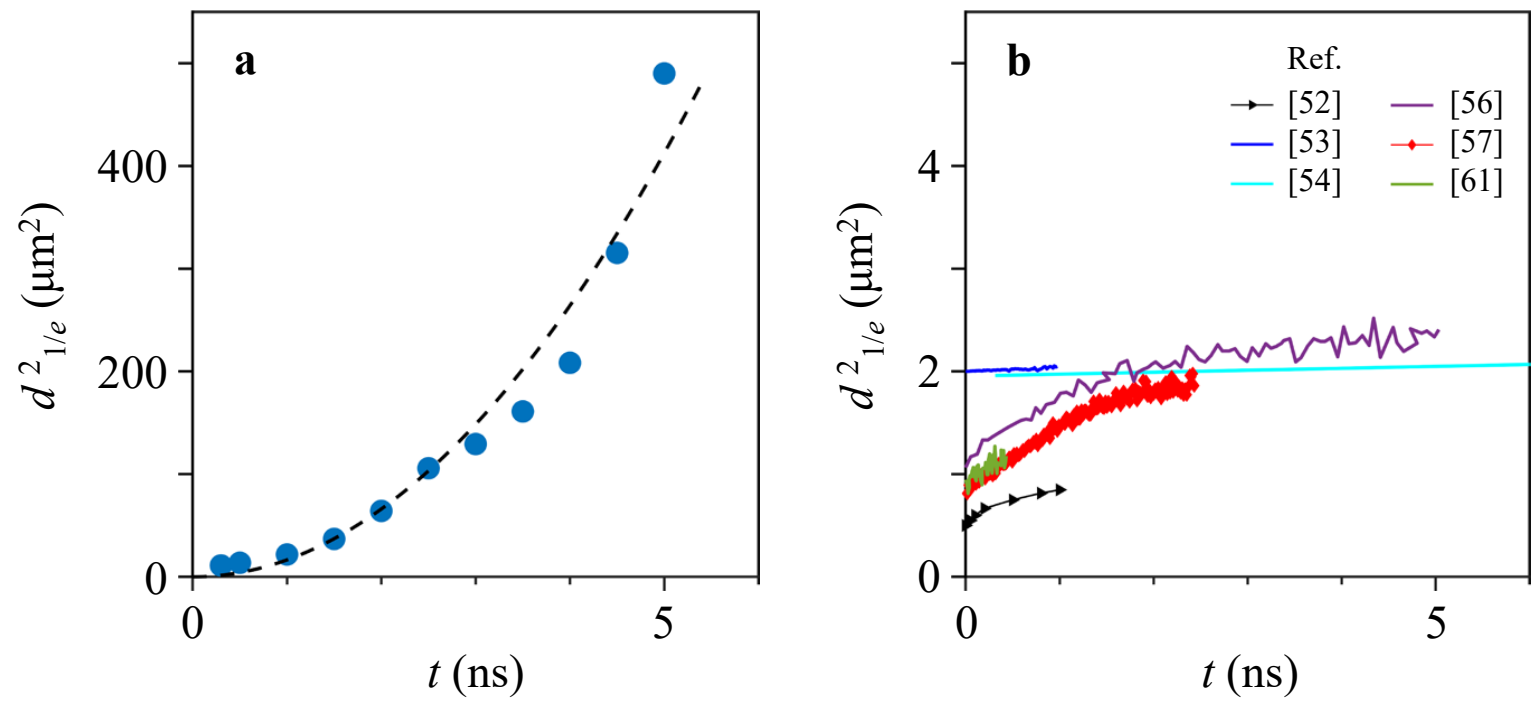

FIG. S12: Comparison of the efficient IX transport kinetics with earlier studies. (a) $d^2_{1/e}(t)$ for the efficient IX transport kinetics (same data for $d_{1/e}(t)$ are shown by blue dots in Fig. 2 in the main text). (b) $d^2_{1/e}(t)$ for IX transport kinetics in earlier studies of vdW TMD HS with Ref. numbers indicating references in the main text. The $1/e$ decay distances of exciton signal $d_{1/e}$ are extracted from figures in these works for the data corresponding to the fastest IX transport kinetics in the excitonic regime in these works. A quadratic growth $d^2_{1/e} \propto t^2$ is shown by dashed line for comparison with the data in (a).

double occupancy [8]. The transport kinetics data are spectrally integrated over the entire IX PL and all IX states are included in the transport measurements.

The shift δE of the lower-energy IX line with P_{ex} is shown in Fig. S11c. This shift δE is used for the mean-field estimate of IX density following Ref. [9] as outlined in SI Section *IX interaction*.

Figure S11 shows that in the regime of the efficient IX transport kinetics ($P_{ex} \sim 500$ μW) and up to the highest densities the IX linewidth is close to the linewidth at the lowest densities. This indicates the excitonic regime for the studied IX transport kinetics. The excitonic regime is consistent with the estimated IX density $n \sim 2 \cdot 10^{11}$ cm^{-2} for the efficient IX transport, significantly smaller than the density of the Mott transition to electron-hole plasma $n_{Mott} > 10^{12}$ cm^{-2} [21, 22]. In contrast, in the plasma regime, the linewidth substantially increases [22].

Comparison with earlier studies of IX transport kinetics in vdW heterostructures

Figure S12a shows $d^2_{1/e}(t)$ for the efficient IX transport kinetics (same data for $d_{1/e}(t)$ are shown by blue dots in Fig. 2 in the main text). Figure S12b shows $d^2_{1/e}(t)$ for IX transport kinetics in earlier studies of vdW TMD HS with Ref. numbers indicating references in the main text. The $1/e$ decay distances of exciton signal $d_{1/e}$ are extracted from figures in these works for the data corresponding to the fastest IX transport kinetics in the excitonic regime in these works. Figure S12 shows the following: (i) For the efficient IX transport kinetics (Fig. S12a), $d_{1/e}$ grows significantly faster than for the IX transport kinetics in earlier studies (Fig. S12b); (ii) In earlier studies (Fig. S12b), $d^2_{1/e}$ grows linearly or sub-linearly with time that is characteristic of diffusion. In contrast, for the efficient IX transport kinetics (Fig. S12a), $d^2_{1/e}$ grows nearly quadratically with time.

A quantitative comparison of IX transport characteristics can be done by comparing the IX mean free time τ_m estimated from the measured IX transport kinetics. For the efficient IX transport kinetics, the IX mobility $\mu \sim 7 \cdot 10^4$ cm^2/eVs at $T \sim 6$ K (Fig. 3c) gives the estimated $\tau_m \sim \mu m \sim 40$ ps. In comparison, for the IX transport kinetics in Ref. [56] in the main text (this work shows fastest IX transport kinetics among the earlier studies, see Fig. S12b), the reported IX diffusivity $D \sim 0.15$ cm^2/s at $T = 4.6$ K gives the estimated $\tau_m \sim Dm/k_B T \sim 0.2$ ps. The estimated IX mean free time for the efficient IX transport kinetics is significantly higher than in earlier studies. The differences between the efficient IX transport kinetics and transport kinetics in earlier studies are discussed in the main text.

References

[1] F. Withers, O. Del Pozo-Zamudio, A. Mishchenko, A.P. Rooney, A. Gholinia, K.Watanabe, T. Taniguchi, S.J. Haigh, A.K. Geim, A.I. Tartakovskii, K.S. Novoselov, Light-emitting diodes by band-structure engineering in van der Waals heterostructures, *Nat. Mater.* **14**, 301 (2015).

[2] L.H. Fowler-Gerace, Zhiwen Zhou, E.A. Szwed, D.J. Choksy, L.V. Butov, Transport and localization of indirect excitons in a van der Waals heterostructure, *Nat. Photonics* **18**, 823 (2024).

[3] Astrid Weston, Yichao Zou, Vladimir Enaldiev, Alex Summerfield, Nicholas Clark, Viktor Zólyomi, Abigail Graham, Celal Yelgel, Samuel Magorrian, Mingwei Zhou, Johanna Zultak, David Hopkinson, Alexei Barinov, Thomas H. Bointon, Andrey Kretinin, Neil R. Wilson, Peter H. Beton, Vladimir I. Fal'ko, Sarah J. Haigh, Roman Gorbachev, Atomic reconstruction in twisted bilayers of transition metal dichalcogenides, *Nat. Nanotechnol.* **15**, 592 (2020).

[4] Matthew R. Rosenberger, Hsun-Jen Chuang, Madeleine Phillips, Vladimir P. Oleshko, Kathleen M. McCreary, Saujan V. Sivaram, C. Stephen Hellberg, Berend T. Jonker, Twist Angle-Dependent Atomic Reconstruction and Moiré Patterns in Transition Metal Dichalcogenide Heterostructures, *ACS Nano* **14**, 4550 (2020).

[5] Shen Zhao, Zhijie Li, Xin Huang, Anna Rupp, Jonas Göser, Ilia A. Vovk, Stanislav Yu. Kruchinin, Kenji Watanabe, Takashi Taniguchi, Ismail Bilgin, Anvar S. Baimuratov, Alexander Högele, Excitons in mesoscopically reconstructed moiré heterostructures, *Nat. Nanotechnol.* **18**, 572 (2023).

[6] Kyle L. Seyler, Pasqual Rivera, Hongyi Yu, Nathan P. Wilson, Essance L. Ray, David G. Mandrus, Jiaqiang Yan, Wang Yao, Xiaodong Xu, Signatures of moiré-trapped valley excitons in $MoSe_2/WSe_2$ heterobilayers, *Nature* **567**, 66 (2019).

[7] Tomasz Woźniak, Paulo E. Faria Junior, Gotthard Seifert, Andrey Chaves, Jens Kunstmann, Exciton g factors of van der Waals heterostructures from first-principles calculations, *Phys. Rev. B* **101**, 235408 (2020).

[8] Zhiwen Zhou, E.A. Szwed, D.J.Choksy, L.H. Fowler-Gerace, L.V. Butov, Long-distance decay-less spin transport in indirect excitons in a van der Waals heterostructure, *Nat. Commun.* **15**, 9454 (2024).

[9] Daijiro Yoshioka, Allan H. MacDonald, Double quantum well electron-hole systems in strong magnetic fields, *J. Phys. Soc. Jpn.* **59**, 4211 (1990).

[10] Akash Laturia, Maarten L. Van de Put, William G. Vandenberghe, Dielectric properties of hexagonal boron nitride and transition metal dichalcogenides: from monolayer to bulk, *npj 2D Mater. Appl.* **2**, 6 (2018).

[11] A.L. Ivanov, Quantum diffusion of dipole-oriented indirect excitons in coupled quantum wells, *Europhys. Lett.* **59**, 586 (2002).

[12] C.J. Dorow, M.W. Hasling, E.V. Calman, L.V. Butov, J. Wilkes, K.L. Campman, A.C. Gossard, Spatially resolved and time-resolved imaging of transport of indirect excitons in high magnetic fields, *Phys. Rev. B* **95**, 235308 (2017).

[13] Matthew P. A. Fisher, Peter B. Weichman, G. Grinstein, Daniel S. Fisher, Boson localization and the superftuid-insulator transition, *Phys. Rev. B* **40**, 546 (1989).

[14] Barbara Capogrosso-Sansone, Şebnem Güneş Söyler, Nikolay Prokof'ev, Boris Svistunov, Monte Carlo study of the two-dimensional Bose-Hubbard model, *Phys. Rev. A* **77**, 015602 (2008).

[15] M. Remeika, M.M. Fogler, L.V. Butov, M. Hanson, A.C. Gossard, Two-dimensional electrostatic lattices for indirect excitons, *Appl. Phys. Lett.* **100**, 061103 (2012).

[16] Fengcheng Wu, Timothy Lovorn, A.H. MacDonald, Theory of optical absorption by interlayer excitons in transition metal dichalcogenide heterobilayers, *Phys. Rev. B* **97**, 035306 (2018).

[17] Hongyi Yu, Gui-Bin Liu, Wang Yao, Brightened spin-triplet interlayer excitons and optical selection rules in van der Waals heterobilayers, *2D Mater.* **5**, 035021 (2018).

[18] Fengcheng Wu, Timothy Lovorn, A.H. MacDonald, Topological Exciton Bands in Moiré Heterojunctions, *Phys. Rev. Lett.* **118**, 147401 (2017).

[19] Hongyi Yu, Gui-Bin Liu, Jianju Tang, Xiaodong Xu, Wang Yao, Moiré excitons: From programmable quantum emitter arrays to spin-orbit-coupled artificial lattices, *Sci. Adv.* **3**, e1701696 (2017).

[20] M. Remeika, J.C. Graves, A.T. Hammack, A.D. Meyertholen, M.M. Fogler, L.V. Butov, M. Hanson, A.C. Gossard, Localization-Delocalization Transition of Indirect Excitons in Lateral Electrostatic Lattices, *Phys. Rev. Lett.* **102**, 186803 (2009).

[21] M.M. Fogler, L.V. Butov, K.S. Novoselov, High-temperature superfluidity with indirect excitons in van der Waals heterostructures, *Nat. Commun.* **5**, 4555 (2014).

[22] Jue Wang, Jenny Ardelean, Yusong Bai, Alexander Steinhoff, Matthias Florian, Frank Jahnke, Xiaodong Xu, Mackillo Kira, James Hone, X.-Y. Zhu, Optical generation of high carrier densities in 2D semiconductor heterobilayers, *Sci. Adv.* **5**, eaax0145 (2019).
