# Supplementary material for: Efficient transport kinetics of indirect excitons in van der Waals heterostructure
Source: Nat Commun. 2026 Sep 26;17:11155. doi: 10.1038/s41467-026-77967-2 (PMC13710747; doi:10.1038/s41467-026-77967-2)
Supplement: Supplementary file 2 [file 41467_2026_77967_MOESM2_ESM.pdf]

Peer Review File

Efficient transport kinetics of indirect excitons in van der Waals heterostructure

Corresponding Author: Professor Leonid Butov

This file contains all reviewer reports in order by version, followed by all author rebuttals in order by version.

Version 0:

Reviewer comments:

Reviewer #1

(Remarks to the Author)

Third report on “Efficient transport kinetics of indirect excitons in van der Waals heterostructure” by Zhiwen Zhou et al.

The authors have done a significant job revising the manuscript and making its main message clearer. Nevertheless, I still see a misleading point in the interpretation of the data.

On the one hand, the authors compare three approaches: purely diffusive (Eq. 1), purely ballistic (Eq. 2), and drift-diffusive (Eq. 3). I appreciate this comparison. It clearly shows that the transport parameters extracted from the reported experiments using basic models fall outside the standard range of parameters expected for such a system.

On the other hand, the authors write: “Therefore, the efficient IX transport kinetics characterized by the anomalously high IX mobility, despite the in-plane disorder, is inconsistent with the diffusive transport” (p. 4, right column, below Fig. 3). They repeatedly stress the point regarding “anomalously high IX mobility” throughout the manuscript, including in the abstract and conclusion. For me—and, I believe, for the audience of Nature Communications—this phrasing would imply that: (i) Eq. (3) is applicable, (ii) it yields high mobility, and (iii) high mobility is equivalent to superfluidity.

However, in fact, superfluidity means that the mobility is infinite, rather than merely finite and large. Equation (3) of the manuscript is nothing but a standard drift-diffusion equation. It is entirely unclear whether it can be applied to a superfluid system. I would assume that the hydrodynamic model for excitons [for example, similar to that presented in Mantsevich and Glazov (Phys. Rev. B 110, 165305, 2024) but extended to a two-fluid scenario (one inviscid and one viscous) should be used for a rigorous analysis of the data. In that case, the viscous part of the excitonic liquid should be described by something similar to Eq. (3) with reasonably small mobility (and reasonably short scattering time), while the superfluid (inviscid) part should expand rapidly as a result of internal pressure from exciton–exciton repulsive interactions (as noted in the aforementioned PRB 2024 paper and also in the Supplementary Information of Ref. [61], Wietek et al.).

I suggest that the authors use more accurate wording regarding the propagation scenario.

An additional unclear point concerns the specific scenario of superfluidity: whether it relates to Bose–Hubbard physics in a moiré system or to the physics of the Berezinskii–Kosterlitz–Thouless transition in a localization-free 2D sample. However, this point can remain an open question for further studies.

Reviewer #2

(Remarks to the Author)
The authors have improved the manuscript through the additional revisions and analyses. The manuscript reports unusually efficient indirect-exciton transport and contains results that may be suitable for publication in Nature Communications. However, several aspects of the interpretation and modeling should be further revised before publication.

1. The superfluidity interpretation should be toned down

The measurements establish rapid IX cloud expansion that deviates strongly from a simple diffusion model. However, the revised analysis does not uniquely distinguish ballistic or superfluid transport from drift-driven or interaction-induced expansion. The superfluidity interpretation should therefore be presented more cautiously and clearly identified as one possible explanation rather than as a conclusion established by the data.

2. The exciton-density calibration should be presented quantitatively in the main text

The relationship among excitation power, exciton density, and moiré filling is central to the comparison with the Bose–Hubbard model. At present, most of the relevant quantitative information is provided only in the Supplementary Information. The main text should include the density-calibration procedure, the assumptions used to convert the measured energy shift into exciton density, the corresponding filling-factor estimate, and an appropriate discussion of the associated uncertainties.

Version 1:

Reviewer comments:

Reviewer #1

(Remarks to the Author)
The authors have tried to provide more accurate wording for the description of their findings. I feel that the manuscript in the current form can be of use for the wide community working on 2D materials, exciton propagation and collective phenomena. I recommend publishing this work.

Reviewer #2

(Remarks to the Author)
The authors have satisfactorily addressed my comments, and I recommend the manuscript for publication.

Open Access This Peer Review File is licensed under a Creative Commons Attribution 4.0 International License, which permits use, sharing, adaptation, distribution and reproduction in any medium or format, as long as you give appropriate credit to the original author(s) and the source, provide a link to the Creative Commons license, and indicate if changes were made.
In cases where reviewers are anonymous, credit should be given to 'Anonymous Referee' and the source.
The images or other third party material in this Peer Review File are included in the article's Creative Commons license, unless indicated otherwise in a credit line to the material. If material is not included in the article's Creative Commons license and your intended use is not permitted by statutory regulation or exceeds the permitted use, you will need to obtain permission directly from the copyright holder.
To view a copy of this license, visit https://creativecommons.org/licenses/by/4.0/

Response to Remarks of Reviewer 1. Reviewer's remarks are marked by italic font, changes in the manuscript are marked by blue font, references correspond to revised manuscript.

Third report on "Efficient transport kinetics of indirect excitons in van der Waals heterostructure" by Zhiwen Zhou et al.

The authors have done a significant job revising the manuscript and making its main message clearer. Nevertheless, I still see a misleading point in the interpretation of the data.

On the one hand, the authors compare three approaches: purely diffusive (Eq. 1), purely ballistic (Eq. 2), and drift-diffusive (Eq. 3). I appreciate this comparison. It clearly shows that the transport parameters extracted from the reported experiments using basic models fall outside the standard range of parameters expected for such a system.

On the other hand, the authors write: "Therefore, the efficient IX transport kinetics characterized by the anomalously high IX mobility, despite the in-plane disorder, is inconsistent with the diffusive transport" (p. 4, right column, below Fig. 3). They repeatedly stress the point regarding "anomalously high IX mobility" throughout the manuscript, including in the abstract and conclusion. For me—and, I believe, for the audience of Nature Communications—this phrasing would imply that: (i) Eq. (3) is applicable, (ii) it yields high mobility, and (iii) high mobility is equivalent to superfluidity.

However, in fact, superfluidity means that the mobility is infinite, rather than merely finite and large. Equation (3) of the manuscript is nothing but a standard drift-diffusion equation. It is entirely unclear whether it can be applied to a superfluid system. I would assume that the hydrodynamic model for excitons [for example, similar to that presented in Mantsevich and Glazov (Phys. Rev. B 110, 165305, 2024) but extended to a two-fluid scenario (one inviscid and one viscous) should be used for a rigorous analysis of the data. In that case, the viscous part of the excitonic liquid should be described by something similar to Eq. (3) with reasonably small mobility (and reasonably short scattering time), while the superfluid (inviscid) part should expand rapidly as a result of internal pressure from exciton–exciton repulsive interactions (as noted in the aforementioned PRB 2024 paper and also in the Supplementary Information of Ref. [61], Wietek et al.).

I suggest that the authors use more accurate wording regarding the propagation scenario.

We use *three approaches* to characterize IX transport and, in particular, determine the range of power densities and temperatures where the efficient IX transport kinetics is observed. Figure 3 shows that all these approaches give a similar range of parameters for the efficient IX transport kinetics that we outline in the text: "All approaches show similar dependence of IX transport kinetics on parameters: The efficient IX transport kinetics characterized by high D, v, and μ (Fig. 3) is observed in a certain range of temperatures and excitation densities and vanishes at high temperatures and at lower or higher excitation densities. The considered approaches quantify the temperature and excitation density dependence of the rapid $d_{1/e}(t)$ enhancement observed in the experiment (Figs. 1b-d)."

We note that these approaches do not aim to provide an accurate description of the efficient IX transport. We clarify this as follows: "For characterization of IX transport kinetics, we use two elemental approaches – in terms of diffusion (Eq. 1) and ballistic transport (Eq. 2), and an approach including drift (Eq. 3). These approaches attribute quantitative characteristics to IX transport such as diffusivity, velocity, and mobility that enables transport comparison at different parameters. We note that these approaches are used for the characterization of IX transport and for rough estimates of the transport characteristics, however, they do not aim to provide an accurate description of the efficient IX transport."

We show that the efficient IX transport kinetics is inconsistent with diffusive transport and show that the efficient IX transport kinetics is qualitatively consistent with superfluidity. First, in section Comparison of

IX transport with diffusive transport, we show that the density and temperature dependence of the efficient IX transport kinetics is inconsistent with diffusive transport: "For diffusive transport comprising both diffusion and drift-diffusion in the HS in-plane disorder potential, IX transport increases both with temperature, due to IX delocalization from local minima of the disorder potential, and with density, due to IX screening of the disorder potential and enhanced interaction-induced drift from the origin [9,12,75]. However, for the efficient IX transport kinetics, a qualitatively different behavior is observed: the efficient IX transport vanishes with temperature and vanishes with density at high densities (Fig. 3), indicating that the efficient IX transport kinetics is inconsistent with the diffusive transport.

Furthermore, both the estimated amplitude of the moiré potential [33-37] and the disorder amplitude outlined above are high. They are significantly higher than the thermal energy at the temperatures where the efficient IX transport kinetics is observed ($k_B T \lesssim 1$ meV, Fig. 3) so the IX thermal energy is insufficient for effective IX delocalization from the local minima of the in-plane potential for the diffusive IX transport [75]. They are also significantly higher than the IX interaction energy at the densities where the efficient IX transport kinetics is observed (the IX energy shift due to the interaction ~ 3 meV for the excitation densities corresponding to the efficient IX transport kinetics, see SI) so the IX interaction energy is insufficient for effective screening of the in-plane potential for the diffusive IX transport [12,16]. Therefore, the efficient IX transport kinetics characterized by the anomalously high IX mobility, despite the substantial in-plane disorder, is inconsistent with the diffusive transport."

Then, in section Comparison of IX transport with BH theory, we show that the density and temperature dependence of the efficient IX transport kinetics is qualitatively consistent with superfluidity: "The efficient IX transport kinetics characterized by the anomalously high IX mobility, despite the substantial in-plane disorder, is qualitatively consistent with the theoretically predicted superfluidity. The BH model predicts the superfluid phase for the number of bosons per lattice site $N \sim 1/2$ and the insulating phase, such as the Mott insulator and the Bose glass, for $N \sim 0$ and $N \sim 1$ [87]. The IX transport enhancement followed by the suppression with density (Fig. 3) is qualitatively consistent with this prediction. The rough estimates also indicate a qualitative agreement with the BH model: For the efficient IX transport kinetics at $P_{ex} \sim 500$ μW (Fig. 3), the energy shift $\delta E \sim 3$ meV (SI); For this energy shift, the mean-field estimate for the IX density [69] gives $n \sim 2 \cdot 10^{11}$ cm^{-2} and $N \sim 1/2$ as outlined in SI. The density ranges for both the measured efficient IX transport and superfluidity in the BH model are broad, and these rough estimates provide qualitative agreement between the measurements and the model. The efficient IX transport kinetics vanishes above $T \sim 10$ K (Fig. 3). This is also qualitatively consistent with the BH theory prediction for superfluidity. A comparison with the BH model is outlined in SI."

This paper presents experimental results. We note that developing a theory of superfluid transport kinetics is beyond the scope of this paper. This is clarified as follows: "The approaches used for the characterization of IX transport in this paper outline the range of parameters where the efficient IX transport kinetics is observed; they do not aim to provide an accurate description of the efficient IX transport kinetics. In particular, developing a theory of superfluid transport kinetics is beyond the scope of this paper."

We avoid a discussion of the model suggested by the Reviewer that is based on the assumption "*superfluidity means that the mobility is infinite*". We use a standard definition of mobility as the ratio of the fluid velocity to the applied energy gradient. It is unclear how the model based on "*superfluidity means that the mobility is infinite*" may comply with this definition as the superfluid velocity is not infinite. For instance, it is limited by the superfluid critical velocity. The problem can be complicated by transient processes of passing the obstacles formed by disorder, vortex formation, etc. The theory of superfluid kinetics in disordered moire potentials is an interesting topic, however, it is beyond the scope of this paper as we clarify: "developing a theory of superfluid transport kinetics is beyond the scope of this paper."

An additional unclear point concerns the specific scenario of superfluidity: whether it relates to Bose–Hubbard physics in a moiré system or to the physics of the Berezinskii–Kosterlitz–Thouless transition in a localization-free 2D sample. However, this point can remain an open question for further studies.

We outline the results in the abstract and, similarly, in the summary: “In this work, we found the efficient IX transport kinetics characterized by anomalously high IX mobility. The efficient IX transport kinetics is observed in the presence of substantial in-plane disorder. The anomalously high IX mobility, its disappearance with increasing temperature, and its non-monotonic dependence on density are qualitatively consistent with theoretical predictions of IX superfluidity.” This wording accurately presents the data and the comparison with theoretical predictions.

Response to Remarks of Reviewer 2. Reviewer's remarks are marked by italic font, changes in the manuscript are marked by blue font, references correspond to revised manuscript.

The authors have improved the manuscript through the additional revisions and analyses. The manuscript reports unusually efficient indirect-exciton transport and contains results that may be suitable for publication in Nature Communications. However, several aspects of the interpretation and modeling should be further revised before publication.

1. The superfluidity interpretation should be toned down

The measurements establish rapid IX cloud expansion that deviates strongly from a simple diffusion model. However, the revised analysis does not uniquely distinguish ballistic or superfluid transport from drift-driven or interaction-induced expansion. The superfluidity interpretation should therefore be presented more cautiously and clearly identified as one possible explanation rather than as a conclusion established by the data.

We use wording that accurately presents the data and the comparison of the data with diffusive transport and with superfluid transport. We show that the efficient IX transport kinetics is inconsistent with diffusive transport and show that the efficient IX transport kinetics is qualitatively consistent with superfluidity. First, in section Comparison of IX transport with diffusive transport, we show that the density and temperature dependence of the efficient IX transport kinetics is inconsistent with diffusive transport: "For diffusive transport comprising both diffusion and drift-diffusion in the HS in-plane disorder potential, IX transport increases both with temperature, due to IX delocalization from local minima of the disorder potential, and with density, due to IX screening of the disorder potential and enhanced interaction-induced drift from the origin [9,12,75]. However, for the efficient IX transport kinetics, a qualitatively different behavior is observed: the efficient IX transport vanishes with temperature and vanishes with density at high densities (Fig. 3), indicating that the efficient IX transport kinetics is inconsistent with the diffusive transport.

Furthermore, both the estimated amplitude of the moiré potential [33-37] and the disorder amplitude outlined above are high. They are significantly higher than the thermal energy at the temperatures where the efficient IX transport kinetics is observed ($k_B T \lesssim 1$ meV, Fig. 3) so the IX thermal energy is insufficient for effective IX delocalization from the local minima of the in-plane potential for the diffusive IX transport [75]. They are also significantly higher than the IX interaction energy at the densities where the efficient IX transport kinetics is observed (the IX energy shift due to the interaction ~ 3 meV for the excitation densities corresponding to the efficient IX transport kinetics, see SI) so the IX interaction energy is insufficient for effective screening of the in-plane potential for the diffusive IX transport [12,16]. Therefore, the efficient IX transport kinetics characterized by the anomalously high IX mobility, despite the substantial in-plane disorder, is inconsistent with the diffusive transport."

Then, in section Comparison of IX transport with BH theory, we show that the density and temperature dependence of the efficient IX transport kinetics is qualitatively consistent with superfluidity: "The efficient IX transport kinetics characterized by the anomalously high IX mobility, despite the substantial in-plane disorder, is qualitatively consistent with the theoretically predicted superfluidity. The BH model predicts the superfluid phase for the number of bosons per lattice site $N \sim 1/2$ and the insulating phase, such as the Mott insulator and the Bose glass, for $N \sim 0$ and $N \sim 1$ [87]. The IX transport enhancement followed by the suppression with density (Fig. 3) is qualitatively consistent with this prediction. The rough estimates also indicate a qualitative agreement with the BH model: For the efficient IX transport kinetics at $P_{ex} \sim 500$ μW (Fig. 3), the energy shift $\delta E \sim 3$ meV (SI); For this energy shift, the mean-field estimate for the IX density [69] gives $n \sim 2 \cdot 10^{11}$ cm^{-2} and $N \sim 1/2$ as outlined in SI. The density ranges for both the measured efficient IX transport and superfluidity in the BH model are broad, and these rough estimates provide qualitative agreement between the measurements and the model. The efficient IX transport kinetics vanishes above T

~ 10 K (Fig. 3). This is also qualitatively consistent with the BH theory prediction for superfluidity. A comparison with the BH model is outlined in SI."

We outline the results in the abstract and, similarly, in the summary: "In this work, we found the efficient IX transport kinetics characterized by anomalously high IX mobility. The efficient IX transport kinetics is observed in the presence of substantial in-plane disorder. The anomalously high IX mobility, its disappearance with increasing temperature, and its non-monotonic dependence on density are qualitatively consistent with theoretical predictions of IX superfluidity." This wording accurately presents the data and the comparison with theoretical predictions.

2. The exciton-density calibration should be presented quantitatively in the main text

The relationship among excitation power, exciton density, and moiré filling is central to the comparison with the Bose–Hubbard model. At present, most of the relevant quantitative information is provided only in the Supplementary Information. The main text should include the density-calibration procedure, the assumptions used to convert the measured energy shift into exciton density, the corresponding filling-factor estimate, and an appropriate discussion of the associated uncertainties.

We *include the exciton-density calibration* in the main text adding *the relevant quantitative information* from *the Supplementary Information* as follows: "The IX out-of-plane dipoles cause repulsive interaction between IXs as shown by the IX studies both in GaAs HS [12,69-82] and vdW HS [56,66,68,83,84]. As shown in Ref. [69], the repulsive interaction between IXs can be approximated by the mean-field formula for the IX energy shift with density $\delta E = nu_0$, $u_0 = 4\pi e^2 d_z/\varepsilon$, where d_z is the separation between the electron and hole layers and ε is the dielectric constant ($d_z \sim 0.6$ nm and $\varepsilon \sim 7.4$ for the HS [85]). This mean-field model [69] is commonly used for estimating IX densities both in GaAs HS and vdW HS, e.g. in Refs. [6,7,11,13,15,31,46,48,56,60,62,66,69,75,82]. We use the mean-free model [69] for density estimates in this work. We note that including correlations lowers u_0 in comparison to the mean-field value, leading to a higher estimated n for a given δE [12,77–79,81,86]. The IX repulsive interaction introduces interaction-induced drift from the origin, adding the term $\nabla[\mu n \nabla(n u_0)]$ to Eq. 1, where μ is the IX mobility [75]."

"The efficient IX transport kinetics characterized by the anomalously high IX mobility, despite the substantial in-plane disorder, is qualitatively consistent with the theoretically predicted superfluidity. The BH model predicts the superfluid phase for the number of bosons per lattice site $N \sim 1/2$ and the insulating phase, such as the Mott insulator and the Bose glass, for $N \sim 0$ and $N \sim 1$ [87]. The IX transport enhancement followed by the suppression with density (Fig. 3) is qualitatively consistent with this prediction. The rough estimates also indicate a qualitative agreement with the BH model: For the efficient IX transport kinetics at $P_{ex} \sim 500$ μW (Fig. 3), the energy shift $\delta E \sim 3$ meV (SI); For this energy shift, the mean-field estimate for the IX density [69] gives $n \sim 2 \cdot 10^{11}$ cm^{-2} and $N \sim 1/2$ as outlined in SI. The density ranges for both the measured efficient IX transport and superfluidity in the BH model are broad, and these rough estimates provide qualitative agreement between the measurements and the model."
